# Nuclear receptor RXRα binds the precursor of miR-103 to inhibit its maturation

**DOI:** 10.1186/s12915-023-01701-3

**Published:** 2023-09-21

**Authors:** Xiaohong Ye, Yun Yang, Jiayue Yao, Mo Wang, Yixin Liu, Guobin Xie, Zhiping Zeng, Xiao-kun Zhang, Hu Zhou

**Affiliations:** 1 School of Pharmaceutical Sciences, Fujian Provincial Key Laboratory of Innovative Drug Target Research, Xiamen University, Xiamen, 361102 Fujian China; 2 High Throughput Drug Screening Platform, Xiamen University, Xiamen, 361102 Fujian China

**Keywords:** Nuclear receptor, RXRα, microRNA, Precursor microRNA, microRNA biogenesis, miR-103, miRNA processing, Exportin-5, Dicer

## Abstract

**Background:**

The maturation of microRNAs (miRNAs) successively undergoes Drosha, Dicer, and Argonaute ˗mediated processing, however, the intricate regulations of the individual miRNA maturation are largely unknown. Retinoid x receptor alpha (RXRα) belongs to nuclear receptors that regulate gene transcription by binding to DNA elements, however, whether RXRα binds to miRNAs to exert physiological functions is not known.

**Results:**

In this work, we found that RXRα directly binds to the precursor of miR-103 (pre-miR-103a-2) via its DNA-binding domain with a preferred binding sequence of AGGUCA. The binding of RXRα inhibits the processing of miR-103 maturation from pre-miR-103a-2. Mechanistically, RXRα prevents the nuclear export of pre-miR-103a-2 for further processing by inhibiting the association of exportin-5 with pre-miR-103a-2. Pathophysiologically, the negative effect of RXRα on miR-103 maturation correlates to the positive effects of RXRα on the expression of Dicer, a target of miR-103, and on the inhibition of breast cancer.

**Conclusions:**

Our findings unravel an unexpected role of transcription factor RXRα in specific miRNA maturation at post-transcriptional level through pre-miRNA binding, and present a mechanistic insight regarding RXRα role in breast cancer progression.

**Supplementary Information:**

The online version contains supplementary material available at 10.1186/s12915-023-01701-3.

## Background

MicroRNAs (miRNAs) are short non-coding RNAs of approximate 20 − 22 nt in length [1]. They are the key post-transcriptional regulators of gene expression and have been implicated in the diverse physiological and pathological processes [2–4]. More and more studies shed light on the biogenesis and maturation of miRNAs. Like protein-coding mRNAs, miRNA genes are initially transcribed by RNA polymerase II into primary miRNAs (pri-miRNAs), the long transcripts containing one or more stem-loop structures [4]. Pri-miRNAs are then processed into precursor miRNA (pre-miRNA) by the Drosha microprocessor complex including DGCR8 and RNA helicases p68 and p72 in the nucleus [5]. The pre-miRNAs are recognized by exportin-5 (XPO5) that mediates their nuclear-cytoplasmic transportation [6]. In the cytoplasm, pre-miRNAs are further processed into a ~ 22 nt mature miRNA-miRNA* duplex by Dicer, a RNase III enzyme [7]. The mature miRNA is then incorporated into the RNA-induced silencing complex (RISC) to be engaged in the silencing of target mRNAs [8]. Although the common mechanisms of miRNA processing are well established, the intricate regulations of the common processing for individual miRNAs need to be extensively explored in order to comprehensively appreciate the biogenesis and functions of miRNAs [9, 10].

Retinoid x receptor alpha (RXRα) belongs to the nuclear receptor family that has a conserved structure including an N-terminal A/B region, a DNA-binding domain (DBD), and a C-terminal ligand-binding domain (LBD) [11, 12]. The DBD is responsible for recognizing and binding the cognate DNA elements, enabling nuclear receptors to specifically regulate gene transcription [13]. Most nuclear receptors including RXRα recognize cis-acting regulator elements of target genes [14–16]. The regulatory elements responding to RXRα contain two or more sequences of AGGTCA, usually arranged in tandem as inverted or direct repeats with variable number of spacing nucleotides [17]. RXRα is able to form homodimer or heterodimer with other nuclear receptors such as retinoic acid receptors (RARs) [18], peroxisome proliferator-activated receptors (PPARs) [19], vitamin D receptor (VDR), and thyroid hormone receptors (TRs) [20], etc. The dimers of RXRα bind to the elements with high affinity, however, compared to other receptors, RXRα displays a relaxed mode of sequence recognition, interacting with only three base-pairs in the element [14].

Recent studies found that transcription factors including some nuclear receptors are capable of binding RNA. RNA aptamer has been shown to interact with estrogen receptor alpha (ERα) to inhibit its transcriptional activity in breast cancer cells [21]. The selected RNA aptamer competes with dsDNA for NF-κB binding [22]. Steroid receptor RNA activator (SRA), a long non-coding RNA, binds to ERα and androgen receptor (AR) with high affinity [23]. Moreover, some transcription factors are involved in miRNA biogenesis and maturation at the post-transcriptional stage. Smads bind to pri-miRNAs and promote Drosha complex-mediated processing of pri-miR-21 into pre-miR-21 [24, 25]. Tumor suppressor p53 enhances the maturation of several miRNAs in response to DNA damage [26]. BRCA1 increases the expression of precursor and mature miRNAs through interacting with Drosha complex [27]. Thus, the intricate regulations of specific miRNA maturation by transcription factors occur under some physiopathological conditions.

In this study, we showed that RXRα bound to pre-miR-103a-2 to inhibit its maturation into miR-103. Moreover, we found that the binding of RXRα prevented pre-miR-103a-2 interacting with and nuclear exporting by XPO5. Finally, we showed the correlation of the inhibitory effect of RXRα on miR-103 maturation with the positive role of RXRα in Dicer expression and the negative role of RXRα in breast cancer cell migration.

## Results

### RXRα directly binds to pre-miR-103a-2

**Fig. 1:**
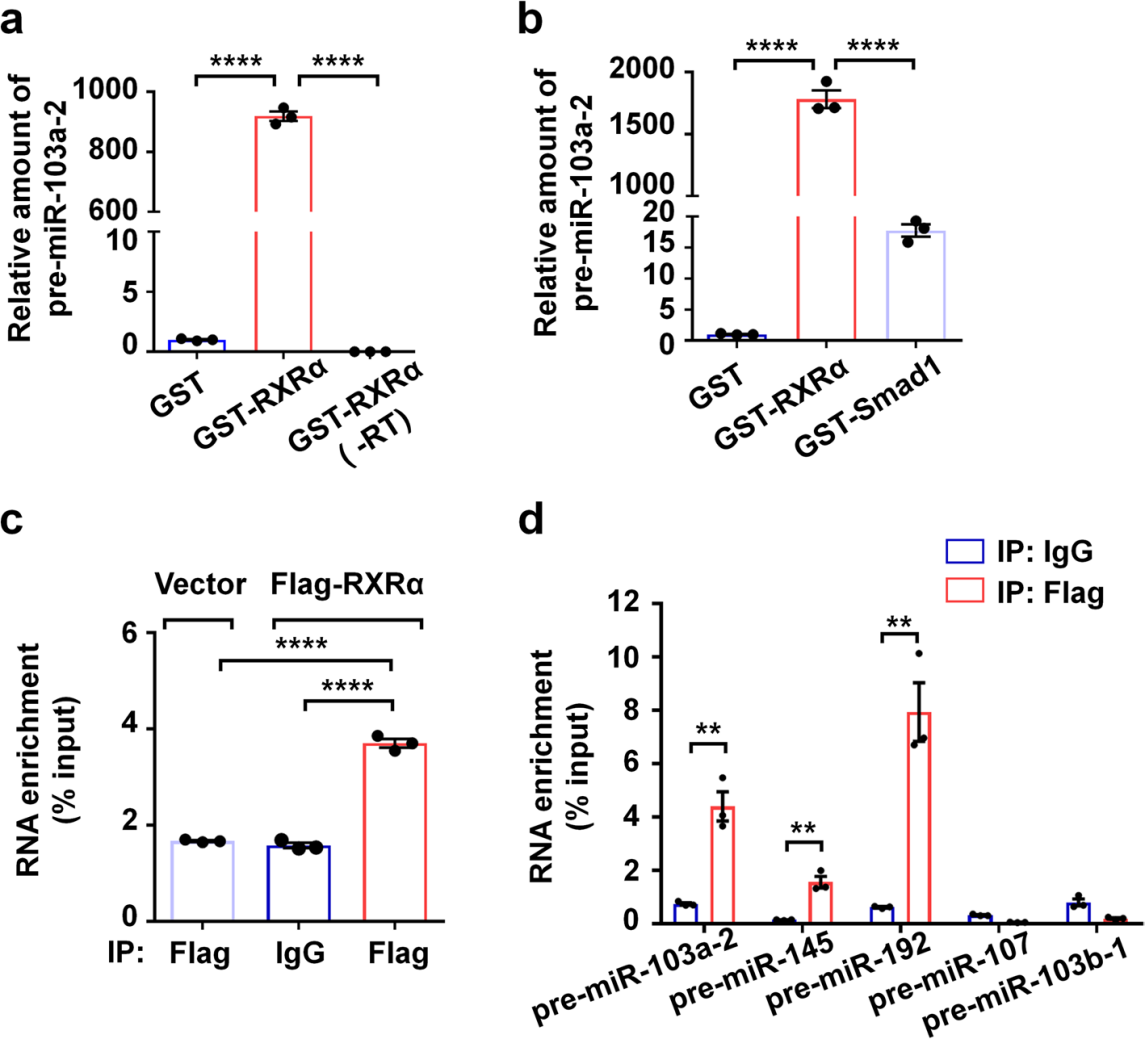
Direct association of RXRα with pre-miR-103a-2. **a** The in vitro transcribed pre-miR-103a-2 was incubated with Glutathione Sepharose beads together with GST or GST-RXRα. The RNA pulled down was eluted and subjected to qRT-PCR analysis to detect pre-miR-103a-2. The group of samples untreated with reverse transcriptase (-RT) but subjected to PCR analysis was a control. The PCR values normalized to the PCR value of pre-miR-103a-2 pulled down by GST were presented as the relative amount of pre-miR-103a-2. **b** GST pull-down analysis of the association of the in vitro transcribed pre-miR-103a-2 with GST, GST-RXRα, and GST-Smad1 proteins. **c** RNA-IP analysis of the association between pre-miR-103a-2 and RXRα in AD293 cells. The lysates from control and stable Flag-RXRα-expressed AD293 cells were subjected to immunoprecipitation with anti-Flag antibody or non-specific IgG, followed by qRT-PCR analysis using the specific primers for pre-miR-103a-2. The amount of pre-miR-103a-2 pulled down was expressed as a percentage of the input. **d** RNA-IP analysis of the association between RXRα and some pre-miRNAs in the stable Flag-RXRα-expressed AD293 cells. Data are presented as mean ± SEM (*n* = 3), ****P < 0.0001, and **P < 0.01

As RXRα binds to retinoid x response element (RXRE) containing AGGTCA sequence with a high affinity [14], we speculated whether RXRα also directly associated with some pre-miRNAs with the corresponding RNA element. We first screened for miRNAs containing AGGUCA sequence from the microRNA database (miRBase), and found that some precursor miRNAs including pre-miR-103a-2 contain the sequence that we termed as R-RXRE. We then investigated whether RXRα could bind to pre-miR-103a-2 by using GST pull-down assay. We found that in vitro transcribed pre-miR-103a-2 was coprecipitated with GST-RXRα fusion protein but not with GST protein (Fig. 1a). Smads that have been reported to bind miR-21 via the consensus sequence CAGACU [24, 25], was used as a control to determine the specificity of RXRα binding. As shown in Additional file 1: Fig. S1a, GST- RXRα and GST-Smad1 were both efficiently pulled down by Glutathione Sepharose beads, however, GST-RXRα coprecipitated with much more pre-miR-103a-2 than did GST-Smad1 (Fig. 1b). These results indicated that RXRα directly binds to pre-miR-103a-2.

We further investigated the interaction of RXRα with pre-miR-103a-2 in cells by using RNA-immunoprecipitation (RNA-IP) assay. Our result showed that Flag antibody was able to pull down more pre-miR-103a-2 in Flag-RXRα stable expression cells than in the control stable cells (Fig. 1c; Additional file 1: Fig. S1b). In Flag-RXRα stable cells, Flag antibody pulled down more pre-miR-103a-2 than did control IgG (Fig. 1c; Additional file 1: Fig. S1b). This result obtained from the cell-based assay could not indicate the direct interaction of RXRα and pre-miR-103a-2, however, it indicated that the interaction occurs in cells. In terms of the direct interaction observed in our GST pull-down assay, it is very likely that the interaction of RXRα and pre-miR-103a-2 in cells should be also direct.

We then investigated the miRNA selectivity of RXRα binding. MiR-107 and miR-103b belong to the same family of miR-103 [28, 29], but their pre-miRNAs do not have R-RXRE. Pre-miR-145 and pre-miR-192 containing R-RXRE were also included for the binding analysis. The result from our RNA-IP assay showed that RXRα robustly bound to pre-miR-103a-2, pre-miR-145, and pre-miR-192, but not to pre-miR-107 or pre-miR-103b-1 (Fig. 1d). These results indicated that the binding of RXRα is of miRNA selectivity, of which the AGGUCA sequence in pre-miRNAs may play an important role.

### RXRα interacts with pre-miR-103a-2 via its DBD

**Fig. 2:**
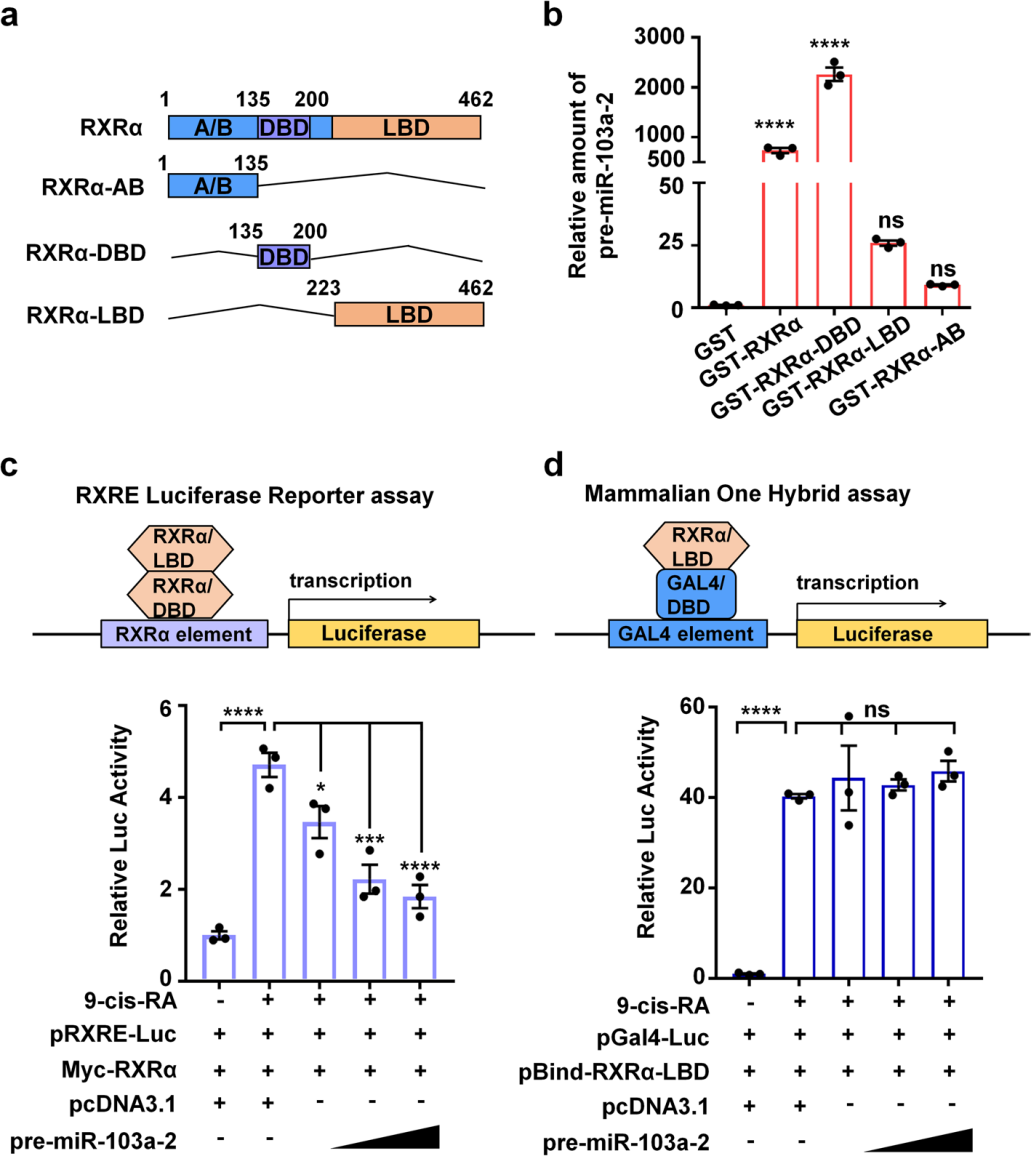
The DBD of RXRα interacts with pre-miR-103a-2. **a** Schematic representation of the structures of the full-length RXRα and its truncated mutants. **b** The in vitro transcribed pre-miR-103a-2 was incubated with bead-immobilized GST, GST-RXRα, or the indicated GST-tagged mutants of RXRα. The RNA pulled down was eluted and subjected to qRT-PCR analysis to detect pre-miR-103a-2. The PCR values normalized to the PCR value of pre-miR-103a-2 pulled down by GST were presented as the relative amount of pre-miR-103a-2. **c,d** HEK293T cells were transfected with pcDNA3.1 or pcDNA3.1-pre-miR-103a-2 plasmids together with pGL6-TA-RXRE-luciferase, renilla and pCMV-Myc-RXRα plasmids (**c**), or together with pG5-luciferase and pBind-RXRα/LBD plasmids (**d**). Cells were then treated with or without 9-cis-RA (10^–7^ M) for 12 h. The value of the firefly luciferase activity was normalized to the renilla luciferase activity to yield the relative luciferase (Luc) activity. Data are presented as mean ± SEM (*n* = 3), ****P < 0.0001, ***P < 0.001, * *P* < 0.05, and ns means not significant

To determine the domains of RXRα responsible for the binding, we constructed recombinant plasmids expressing GST-tagged truncated mutants of RXRα (Fig. 2a; Additional file 1: Fig. S1c). The data from our GST pull-down assay showed that the GST-tagged full-length RXRα and DBD truncated mutant, but not the GST-tagged AB or LBD mutants significantly pulled down pre-miR-103a-2 in vitro (Fig. 2b). This result indicated that the DBD is responsible for RXRα binding to pre-miR-103a-2.

9-cis-retionic acid (9-cis-RA), an agonist of RXRα, binds to the LBD of RXRα and activates RXRα’s transcriptional activity [30]. Consistently, we found that 9-cis-RA strongly induced RXRα-mediated activation of RXRE-luciferase reporter, reflecting that RXRα bound to RXRE and 9-cis-RA induced RXRα transactivation (Fig. 2c). We found that the effect of 9-cis-RA on activating the reporter was substantially inhibited by pre-miR-103a-2 in a dose-dependent manner (Fig. 2c). This result suggested that pre-miR-103a-2 competes with RXRE for RXRα binding. Notably, we did not observe significant effect of pre-miR-103a-2 on 9-cis-RA-induced transactivation of the chimeric protein Gal4/DBD-RXRα/LBD (a fusion protein with the DBD of Gal4 fused to the LBD of RXRα) in our mammalian one-hybrid assay (Fig. 2d). The transactivation of RXRα and Gal4/DBD-RXRα/LBD respectively depended on RXRα/DBD and Gal4/DBD binding to their cognate DNA elements (Fig. 2c, d). Thus, these suggested that pre-miR-103a-2 inhibits RXRα/DBD but not Gal4/DBD binding to the corresponding cognate DNA elements, likely due to the selective binding of pre-miR-103a-2 with RXRα/DBD.

### R-RXRE is important for pre-miR-103a-2 binding to RXRα

**Fig. 3:**
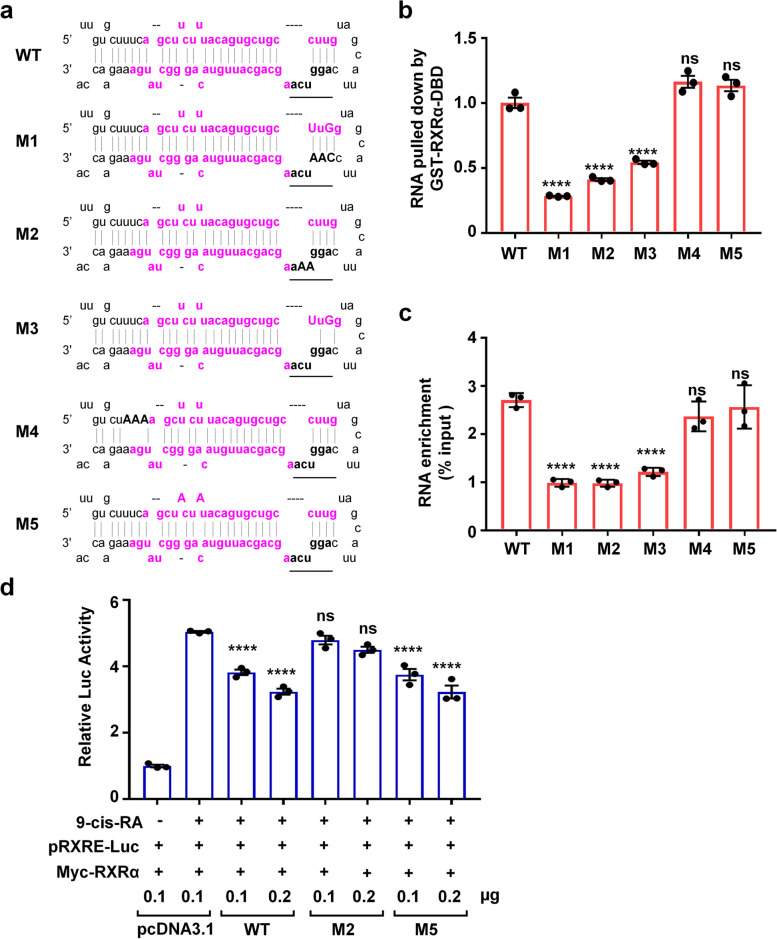
R-RXRE is important for pre-miR-103a-2 binding to RXRα. **a** Schematic diagram of pre-miR-103a-2 wild-type (WT) and mutants (M1–M5). The R-RXRE is underlined and in black, and the sequences of the mature miRNAs are in magenta. The substituting bases are shown in bold capital letters. **b** In vitro transcribed pre-miR-103a-2 and its mutants were pulled down by the recombinant GST-RXRα/DBD or GST protein followed by quantifying by qRT-PCR. The PCR values of GST-RXRα/DBD fusion protein was normalized to that of GST protein. **c** RNA-IP analysis of the association of Flag-RXRα with pre-miR-103a-2 and its mutants in Flag-RXRα stable AD293 cells. **d** HEK293T cells were transfected with pGL6-TA-RXRE-luciferase and Myc-RXRα expression plasmids together with pre-miR-103a-2 or its mutant expression plasmids for 24 h. Cells were then treated with or without 9-cis-RA (10^–7^ M) for 12 h. The value of the firefly luciferase activity was normalized by the renilla luciferase activity to generate the relative luciferase (Luc) activity. Statistical significance of the difference between the second group and the other groups are shown. Data are presented as mean ± SEM (*n* = 3), ****P < 0.0001, and ns means not significant

Previous studies showed that RXRα is able to bind to RXRE as a monomer, or as a homodimer with a higher specificity and affinity [31]. We showed above that R-RXRE sequence AGGUCA might be required for RXRα binding to pre-miRNAs (Fig. 1d). To confirm this, we performed the binding assays by using pre-miR-103a-2 mutants. We introduced 2 − 5 nt mutations in pre-miR-103a-2 (Fig. 3a), of which mutants M1, M2 and M3 were of point mutations in the R-RXRE sequence while mutants M4 and M5 were of point mutations in the 5′ terminal of pre-miR-103a-2. Compared with wild-type pre-miR-103a-2, mutants M1, M2 and M3 but not M4 or M5 showed reduced binding of RXRα/DBD in our GST pull-down assay (Fig. 3b). Consistently, our RNA-IP assay showed that mutants M1, M2 and M3 but not M4 or M5 were less coprecipitated with RXRα than was wild-type pre-miR-103a-2 (Fig. 3c).

Structural predictions showed that compared with wild-type pre-miR-103a-2, the secondary structures of R-RXRE and its adjacent sequences in M1 and M3 but not in M2, M4 or M5 are significantly altered (Additional file 1: Fig. S2). M4 has a significant structural alteration in its stem area. We speculated that the secondary structure change even without affecting RXRα binding may affect the mature procedures of pre-miR-103a-2, e.g. the recognition and binding of XPO5 and the processing by Dicer complex, which would complicate our investigation of the interaction of RXRα and pre-miR-103a-2. Therefore, we selected mutants M2 and M5 without significant structural change for further study. We first compared their ability of inhibiting RXRα transactivation with wild-type pre-miR-103a-2 by using RXRE reporter assay. Whereas wild-type pre-miR-103a-2 and M5 mutant dose-dependently inhibited 9-cis-RA-induced RXRα transactivation, M2 mutant did not show significant effect (Fig. 3d), indicating that wild-type pre-miR-103a-2 and M5 mutant but not M2 mutant competed with RXRE for RXRα binding. Together, these results revealed that the intact R-RXRE is essential for RXRα binding to pre-miR-103a-2.

### RXRα inhibits the processing of pre-miR-103a-2 into miR-103

**Fig. 4:**
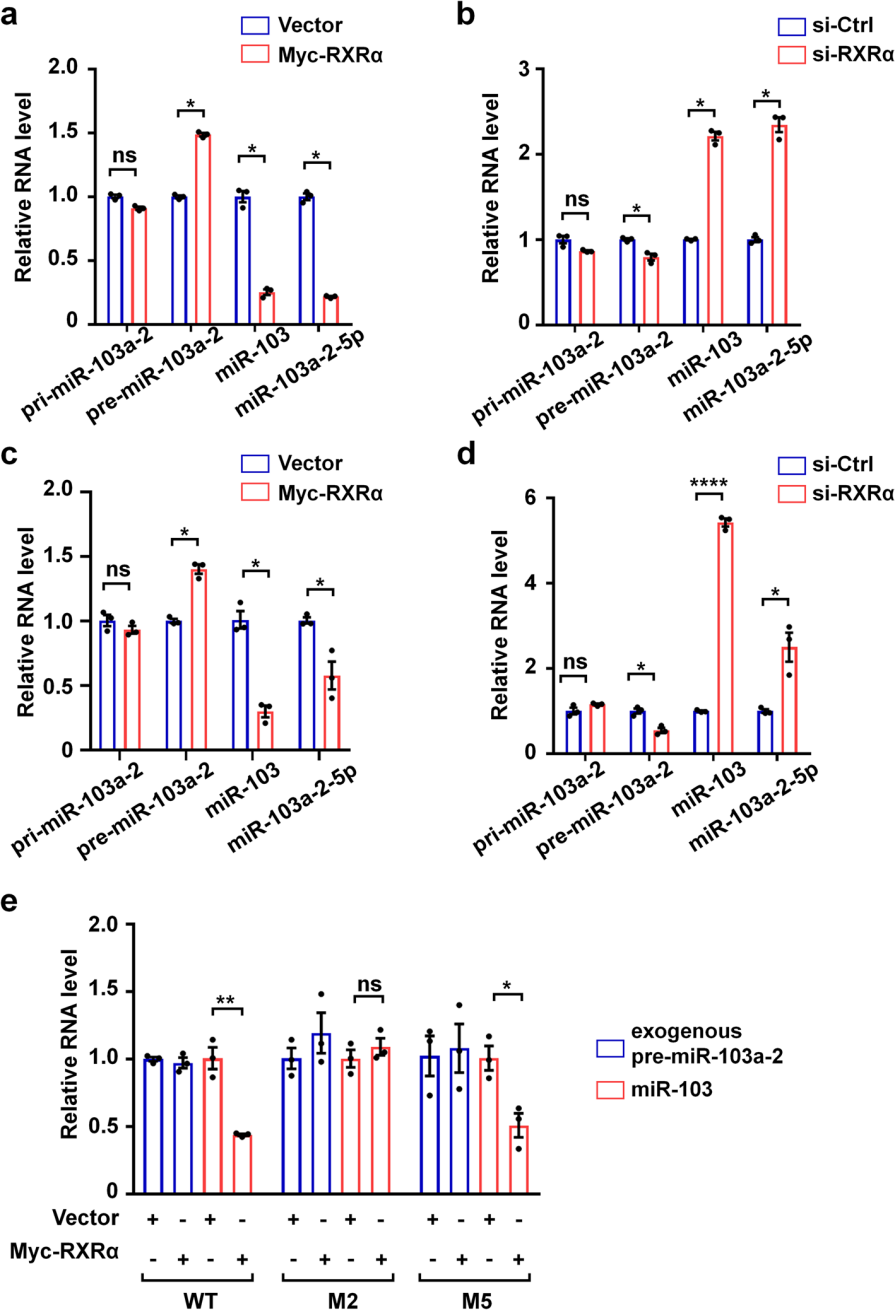
RXRα inhibits the processing of pre-miR-103a-2 into miR-103. **a**–**d** HeLa cells (**a**,**b**) or MDA-MB-231 cells (**c**,**d**) were transfected with the indicated expression plasmids or siRNAs for 48 h. QRT-PCR was applied to detect the expression levels of the indicated primary (pri), precursor (pre), and mature forms of miRNAs. The values of the groups of Myc-RXRα overexpression and RXRα knockdown were normalized to their corresponding control groups, respectively. **e** HeLa cells were transfected with pCMV vector or pCMV-Myc-RXRα plasmid, along with pcDNA3.1-pre-miR-103a-2 plasmid and pcDNA3.1 vectors containing M2 and M5 genes, and the relative RNA levels of pre-miR-103a-2 and miR-103 were analyzed. To exclude the interference due to the transfection efficiency, neomycin gene in the pcDNA3.1 vector but not in the pCMV vector was used as an internal control for normalization. Data are presented as mean ± SEM (*n* = 3), ****P < 0.0001, **P < 0.01, * *P* < 0.05, and ns means not significant

To reveal the physiological function of RXRα binding to pre-miR-103a-2, we investigated whether RXRα regulated the mature processing of pre-miR-103a-2. To this end, the effects of RXRα on the expression levels of primary, precursor, and mature forms of miR-103 in the human cervical cancer cell line HeLa were evaluated by using quantitative real-time reverse transcription PCR (qRT-PCR). MiR-103 (also named as miR-103a-3p) and miR-103a-2-5p (also named as miR-103-2*) are two mature forms of pre-miR-103a-2 after processing. Overexpression of RXRα in HeLa cells did not significantly alter the expression level of pri-miR-103a-2, but increased the expression of pre-miR-103a-2 and decreased the expression of miR-103 and miR-103a-2-5p (Fig. 4a). Knockdown of RXRα in HeLa cells attenuated the expression of pre-miR-103a-2 and upregulated the expression of miR-103 and miR-103a-2-5p, whereas the expression of pri-miR-103a-2 was not significantly affected by knockdown of RXRα (Fig. 4b). Similar results were obtained in the breast cancer cell line MDA-MB-231 (Fig. 4c, d). These results indicated that RXRα may inhibit the processing from pre-miR-103a-2 to miR-103, but not from pri-miR-103a-2 to pre-miR-103a-2.

We further evaluated the requirement of the association of RXRα and pre-miR-103a-2 in RXRα’s regulation of miR-103 processing by using the mutants of pre-miR-103a-2. Similarly, the processing of miR-103 from ectopic pre-miR-103a-2 was significantly decreased in HeLa cells transfected with Myc-RXRα (Fig. 4e). RXRα also showed inhibitory effect on the processing of M5 mutant that bound to RXRα, but not of M2 mutant that did not bind to RXRα (Fig. 4e). Together, these results indicated that RXRα binds to pre-miR-103a-2 to inhibit its processing into miR-103.

### RXRα inhibits XPO5-mediated nuclear export of pre-miR-103a-2

**Fig. 5:**
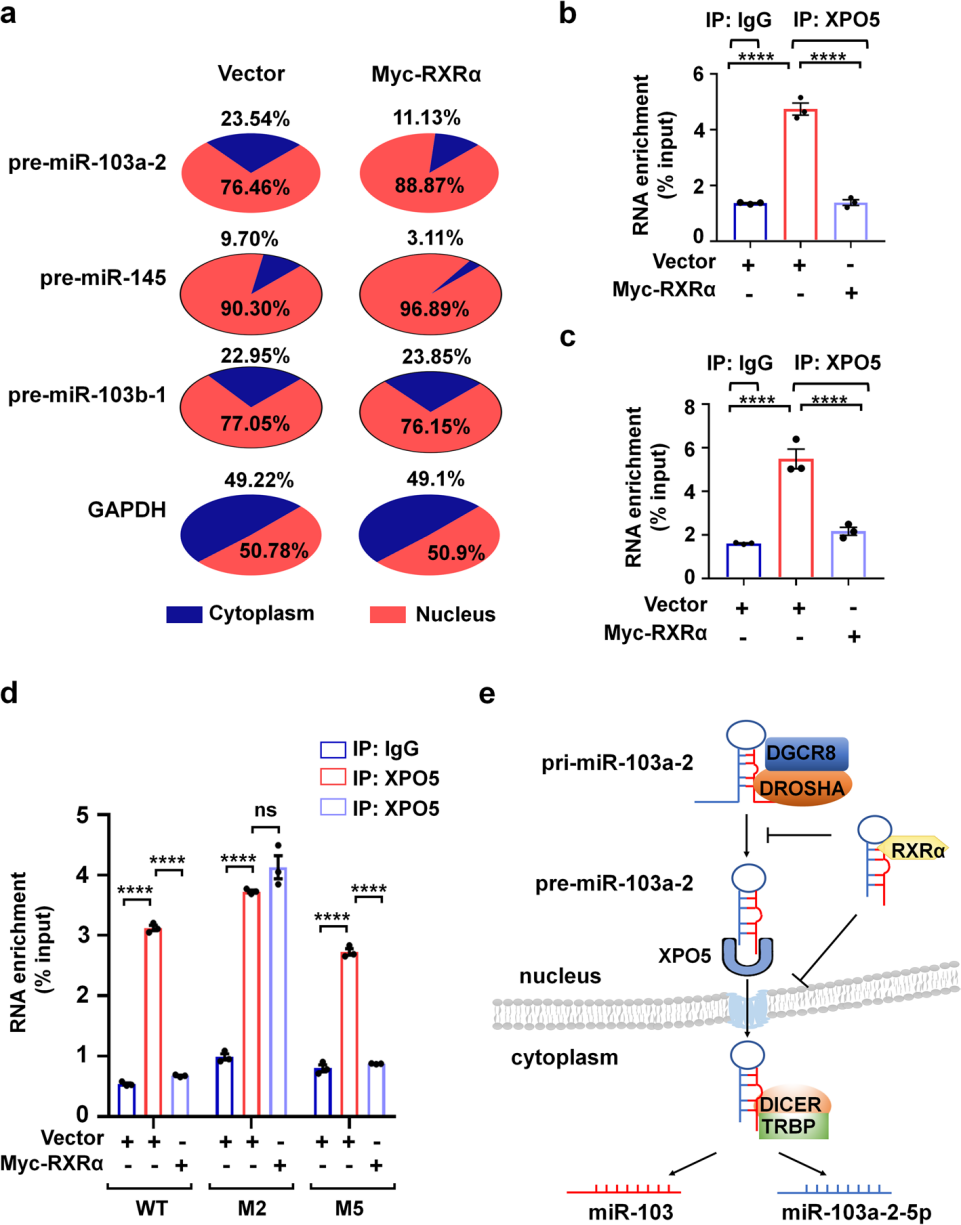
RXRα inhibits XPO5-mediated nuclear export of pre-miR-103a-2. **a** HeLa cells transfected with control or Myc-RXRα expression plasmids were subjected to cellular fractionation to obtain nucleus and cytoplasm parts for RNA extraction. The expression levels of pre-miR-103a-2, pre-miR-145, pre-miR103b-1 and *GAPDH* mRNA in nucleus and cytoplasm were quantified by qRT-PCR. **b,c** HeLa cells (**b**) or MDA-MB-231 cells (**c**) were transfected with control or Myc-RXRα expression plasmids. Cell lysates was used for immunoprecipitation with the anti-XPO5 antibody or non-specific IgG, and the immunoprecipitated pre-miR-103a-2 was quantified by qRT-PCR. **d** HeLa cells were transfected with Myc-RXRα expression plasmid along with pre-miR-103a-2 or its mutant expression plasmids. Cell lysate was used for immunoprecipitation with the anti-XPO5 antibody or non-specific IgG, and the immunoprecipitated pre-miR-103a-2 and its mutants were quantified by qRT-PCR. **e** A mechanistic working model of RXRα inhibiting pre-miR-103a-2 processing. Data are presented as mean ± SEM (*n* = 3), ****P < 0.0001, and ns means not significant

We further explored the underlying mechanism of RXRα function in the inhibition of miR-103 processing. As previous report that RXRα mainly resides in the nucleus [15], it was conceivable that the association of RXRα with pre-miR-103a-2 occurred in the nucleus. The processing of pre-miRNAs to mature miRNAs requires the transportation of pre-miRNAs from nucleus to cytoplasm where Dicer mediates its further maturation. We thus hypothesized that RXRα prevented the nuclear-cytoplasmic transportation of pre-miR-103a-2. To test it, we performed the cytoplasm and nucleus fraction assay to determine whether RXRα affected the subcellular distribution of pre-miR-103a-2. The efficiency of our cellular fractionation assay was confirmed by western blot analysis with an antibody specific for the cytoplasmic α-tubulin protein and an antibody specific for the nuclear PARP protein (Additional file 1: Fig. S3a). We found that the distribution of pre-miR-103a-2 in the nucleus was much elevated by RXRα in HeLa cells (from 76.46% to 88.87%) (Fig. 5a). Meanwhile, we also analyzed the distribution of pre-miR-145 capable of binding RXRα and pre-miR-103b-1 incapable of binding RXRα. As expected, pre-miR-145, similar to pre-miR-103a-2, retained more in the nucleus in RXRα overexpressed cells than in control cells, whereas the distribution of pre-miR-103b-1 was not significantly affected by RXRα overexpression (Fig. 5a).

XPO5 is a dsRNA-binding karyopherin protein that mediates the nuclear export of pre-miRNAs [32, 33]. We found that the association of XPO5 and pre-miR-103a-2 was significantly inhibited by RXRα overexpression both in HeLa cells and MDA-MB-231 cells (Fig. 5b, c; Additional file 1: Fig. S3b). Notably, RXRα also inhibited the association of XPO5 with M5 mutant but not with M2 mutant (Fig. 5d), likely due to the binding of RXRα with M5 but not with M2 mutant. Together, these results suggested that the binding of RXRα inhibits the association of pre-miR-103a-2 with XPO5, thereby preventing pre-miR-103a-2 nuclear export for cytoplasmic maturation (Fig. 5e).

### The correlation of the effects of RXRα on breast cancer cell migration and miR-103 processing

**Fig. 6:**
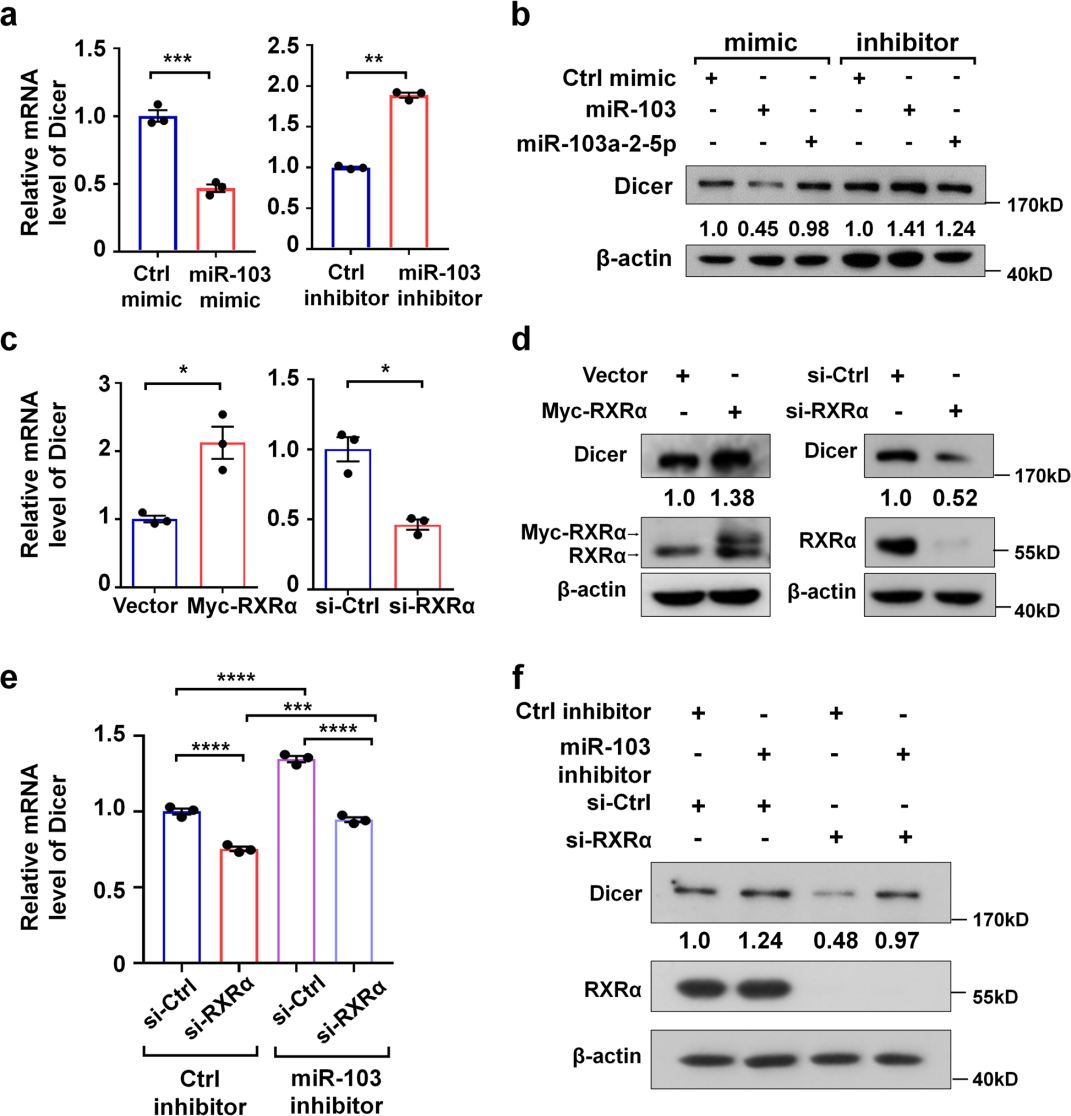
RXRα up-regulates the expression level of Dicer. **a**, **b** The mRNA and protein levels of Dicer in MDA-MB-231 cells transfected with the indicated miRNA mimics or inhibitors were analyzed by qRT-PCR assay (**a**) and western blot assay (**b**), respectively. **c**,** d** The mRNA and protein levels of Dicer in MDA-MB-231 cells transfected with Myc-RXRα expression plasmid or RXRα siRNA (si-RXRα) were analyzed by qRT-PCR assay (**c**) and western blot assay (**d**), respectively. **e,f** MDA-MB-231 cells were transfected with RXRα siRNA or control siRNA along with miR-103 inhibitor or control inhibitor. The mRNA and protein levels of Dicer were analyzed by qRT-PCR assay (**e**) and western blot assay (**f**), respectively. The intensity of the bands of Dicer and β-actin was quantified by ImageJ software, and the intensity ratios of Dicer/β-actin were presented (**b**, **d**, and **f**). Ctrl, control. Data are presented as mean ± SEM (*n* = 3), ****P < 0.0001, ***P < 0.001, ** *P* < 0.01, and *P < 0.05

It has been reported that miR-103 is highly expressed in triple-negative breast cancers [34, 35], promoting breast cancer invasion and metastasis by targeting Dicer [36]. Consistently, we found that transfection of miR-103 mimic and inhibitor respectively reduced and increased the mRNA level of Dicer in MDA-MB-231 cells (Fig. 6a). The mimic and inhibitor of miR-103 but not of miR-103a-2-5p, also down-regulated and up-regulated the protein level of Dicer, respectively (Fig. 6b). These results confirmed the previous report that Dicer is the target of miR-103 [36]. Interestingly, overexpression of RXRα increased the mRNA and protein levels of Dicer, while knockdown of RXRα decreased the mRNA and protein levels of Dicer (Fig. 6c, d). Thus, the opposite effects of miR-103 and RXRα on Dicer expression likely reflects the negative regulation of RXRα in miR-103 generation. Furthermore, our rescue experiment showed that the down-regulation of Dicer mRNA and protein by RXRα knockdown was prevented by miR-103 inhibitor (Fig. 6e, f). Together, these results indicated that RXRα up-regulates the expression of Dicer by inhibiting miR-103 generation.

**Fig. 7:**
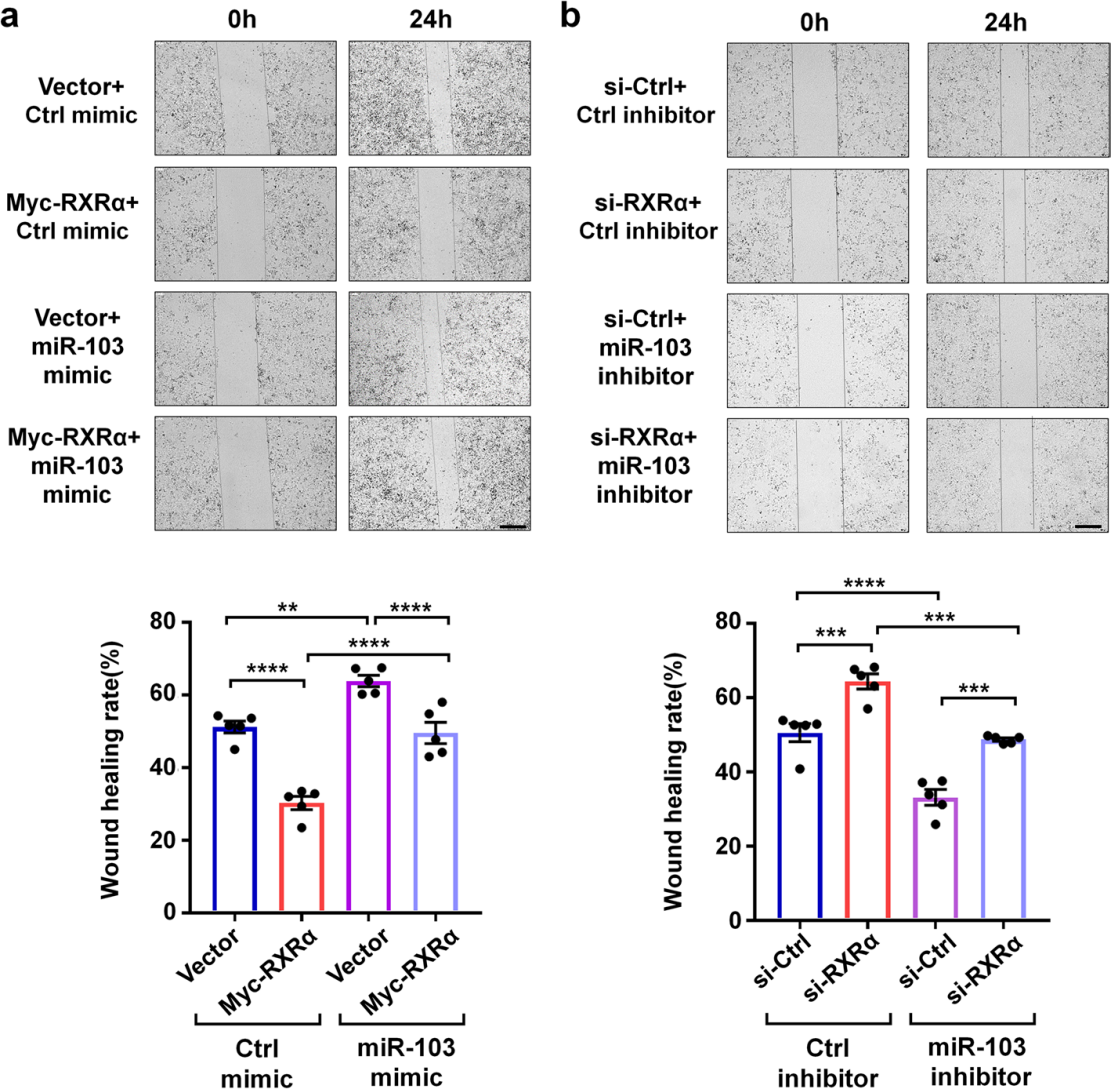
RXRα and miR-103 reciprocally compromise each effect on MDA-MB-231 cell migration. Wound healing assays were performed in MDA-MB-231 cells transfected with Myc-RXRα expression plasmid and/or miR-103 mimic (**a**), or in MDA-MB-231 cells transfected with RXRα siRNA and/or miR-103 inhibitor (**b**). The wound healing areas were quantified by ImageJ software. Scale bar, 50 μm. Data are presented as mean ± SEM (*n* = 5), ****P < 0.0001, ***P < 0.001, and ** *P* < 0.01

Transfection of miR-103 mimic and inhibitor respectively promoted and inhibited MDA-MB-231 cell migration (Fig. 7a, b), consistent with the previous reports that miR-103 is a promoter of breast cancer [35, 36]. On the contrary, overexpression of RXRα significantly inhibited MDA-MB-231 cell migration (Fig. 7a), while knockdown of RXRα by small interfering RNA promoted cell migration (Fig. 7b). Importantly, the effects of RXRα overexpression and miR-103 mimic on cell migration were compromised each other, while RXRα knockdown and miR-103 inhibitor reciprocally compromised each effect on cell migration as well (Fig. 7a, b). Together, these results suggested that RXRα inhibits cancer cell migration by down-regulating miR-103.

## Discussion

The composition and structure of DNA and RNA are similar. If a protein is capable of binding to DNA, it is likely inclined to bind to RNA as well. Indeed, some studies showed that several transcription factors, including TFIIIA, Stat1, p53, and Smads with DNA binding capability, also bind to RNA [37]. Here, we presented another example. We showed that nuclear receptor RXRα, also being a transcription factor, directly bound to pre-miR-103a-2. Thus, it seems that the binding of RNA is prevalence among the transcription factors. The association of transcription factors and DNA is mediated by the DBD and the cognate DNA elements [19, 38]. Interestingly, we found that the DBD was also responsible for RXRα binding to pre-miR-103a-2. Moreover, we found that the cognate element of DNA and RNA for RXRα binding may share the similar sequence AGGT(U)CA. Similar conclusion was obtained from the study of Smads binding to pri-miRNA [24, 25]. Thus, it seems that the transcription factors employ the similar modes for binding to both DNA and RNA.

The general procedure and mechanism of miRNA maturation from pri-miRNA is well established [10, 39]. However, it is unlikely that the maturation rates of all miRNAs are equal in tissues and cells [40]. Rather, in some specific context, cells need speed up or slow down the maturation rates of specific miRNAs to meet the requirements of selective gene expression [7, 39]. Thus, cells need devise delicate mechanisms to selectively regulate the processing of specific miRNAs. It has been reported that p53 and Smads bind to specific RNA sequence or structure and regulate a subset of miRNAs processing [24, 26]. Similarly, we found that RXRα regulated the processing of miR-103 maturation, providing a regulation mechanism of specific miRNA processing. As a nuclear receptor, the function of RXRα is tightly regulated by its ligands [11, 15, 30, 41, 42]. It should be interesting to know whether the ligands regulate the effect of RXRα on miR-103 processing.

The compartmentalization of molecules in the subcellar structures generally defines the functions of the molecules to some extent. The nuclear distribution of RXRα enables it binding to DNA to exert its transcriptional activity [15, 43]. The processing of pri-miRNAs to pre-miRNAs takes place in the nucleus because of the nuclear localization of the Drosha complex – the processing machinery. The further processing from pre-miRNAs to mature miRNAs occurs in the cytoplasm because both the Dicer complex and the Argonaute complex localize in the cytoplasm. Thus, XPO5-mediated nuclear export of pre-miRNAs is an essential step in miRNA maturation. We found that the binding of RXRα inhibited the association of pre-miR-103a-2 with XPO5, thereby preventing the nuclear-cytoplasmic transportation of pre-miR-103a-2. Thus, this mechanism of miR-103 inhibition by RXRα depended on their interaction. If the interaction can be regulated by some stimuli, such as the ligands of RXRα, the RXRα-mediated processing of miR-103 is thereby regulatable.

The influence of the interaction to the activities of the two interacting molecules should be reciprocal. It is conceivable that pre-miR-103a-2 may inhibit RXRα binding to its cognate DNA elements. Indeed, we found that pre-miR-103a-2 dose-dependently inhibited 9-cis-RA-induced activation of RXRE reporter. This suggested that pre-miR-103a-2 competed with RXRE for association with RXRα. Notably, this result indicated that pre-miR-103a-2 regulated the transcriptional activity of RXRα. However, what is the selectivity and magnitude of the regulation need be further explored.

It has been reported that RXRα is down-regulated in thyroid cancer [44], stomach cancer [45] and non-small-cell lung cancer [46], indicating that RXRα may function as a tumor suppressor. Here, we also showed that RXRα was a suppressor in breast cancer progression, indicating from its negative role in the migration of MDA-MB-231 breast cancer cells. It has been reported that miR-103 confers tumor cell migrating capacity by targeting Dicer [36]. We found that RXRα upregulated the expression of Dicer, likely through its down-regulation of miR-103 biogenesis. As miRNA has multi-targets, RXRα may affect other target genes of miR-103 to exert its anti-breast cancer effects, which need further investigation.

## Conclusions

In summary, we demonstrated that the direct association of nuclear receptor RXRα with a preferred binding sequence of AGGUCA prevents pre-miR-103a-2 nuclear-cytoplasmic exporting by XPO5, resulting in the inhibition of pre-miR-103a-2 maturation into miR-103 and miR-103-mediated downregulation of Dicer in breast cancers. Thus, RXRα is a multifunctional gene-expression regulator that modulate gene expression transcriptionally through DNA binding as a transcription factor and post-transcriptionally through pre-miRNA binding as a miRNA processing regulator.

## Methods

### Reagents and antibodies

Anti-RXRα (D20, sc-553, 1:500), anti-Dicer (H-212, sc-30226, 1:1000), anti-Exportin 5 (A-11, sc-271036, 1:1000), anti-α-tubulin (B-7, sc-5286, 1:1000), and normal mouse IgG (sc-2025) antibodies were from Santa Cruz Biotechnology (Santa Cruz, CA, USA). Anti-β-actin (A5441, 1:2000) and anti-Flag (F1804, 1:2000) antibodies, and 9-cis-RA (R4643) were from Sigma-Aldrich (St. Louis, MO, USA). Anti-PARP (Cat. 9542, 1:500) antibody was from Cell Signal Technology (Beverly, MA, USA). Goat anti-mouse (111–035-003, 1:5000) and goat anti-rabbit (115–035-003, 1:5000) lgG-HRP-conjugated were purchased form the Jackson ImmunoResearch. Protein G Plus-Agarose Suspension (IP04) was from Millipore. Small-interfering RNAs against human RXRα (SASI_Hs01_00097638, SASI_Hs01_00097639, SASI_Hs01_00097640) and control siRNA (SIC001) were from Sigma-Aldrich. MiR-103 mimic (miR10000101-1–5), miR-103a-2-5p mimic (miR10009196-1–5), control microRNA mimic (miR1N0000002-1–1), miR-103 inhibitor (miR20000101-1–5), miR-103a-2-5p inhibitor (miR20009196-1–5), and control microRNA inhibitor (miR2N0000002-1–1) were from RiboBio (Guangzhou, China).

### Plasmid constructions

Pre-miRNAs containing about 50 nt flanking sequence and restriction enzyme sites were synthesized into pUC57 vector and then cloned into pcDNA3.1( +) vector. Mutants of pre-miR-103a-2 were constructed by using Phusion™ Site-Directed Mutagenesis kit (Thermo Fisher Scientific, Cat. F541) and cloned into pcDNA3.1 vector. GST-RXRα and Myc-RXRα expression plasmids were cloned into pGEX-4 T-2 and pCMV-Myc vector respectively, which have been used in our previous papers [47, 48]. GST-Smad1 and the mutants of GST-RXRα-AB, GST-RXRα-DBD and GST-RXRα-LBD were cloned into pGEX-4T-2 vector.

### Cell culture

MDA-MB-231, AD293 and HEK293T cells were purchased from the American Type Culture Collection (Manassas, VA, USA) and cultured in DMEM with 10% fetal bovine serum. HeLa cells were purchased form Cell Bank of Chinese Academy of Sciences and maintained in MEM containing 10% fetal bovine serum. All cells were cultured in a humidified atmosphere containing 5% CO_2_ at 37 ℃.

### Cell transfection

Cell transient transfection was carried out with Lipofectamin™ 2000 (Invitrogen) for MDA-MB-231 and HeLa cells or with polyethylenimine (PEI) for HEK293T cells. The amount ratio of the transfection reagent and plasmid was 2 μL to 1 μg, while the amount ratio of the transfection reagent and small RNAs was 1 μL to 20 μmol. The transfection reagents and DNAs/RNAs were diluted with Opti-MEM™ (Gibco), respectively, followed by incubation for 5 min. The diluted DNAs/RNAs solution and the diluted transfection reagent were mixed. After incubation for 20 min, the mixed solution was then added to the cell culture medium for transfection, and the medium was changed 6—8 h later. Cells were harvested 36 or 48 h after transfection. The amount of DNA for transfection of 10^5^ cells is 100 ng. The final transfection concentration of small-interfering RNAs and miRNA mimics was 50 nM, and the final transfection concentration of miRNA inhibitors was 100 nM. AD293 cells transfected with pcDNA3.1 vector and pcDNA3.1-Flag-RXRα plasmid were selected by G418 (1 mg/mL) for 3 weeks. The colonies of singe cell clone were picked up and cells were expanded to obtain the stable cell lines.

### RNA extraction and quantitative real-time polymerase chain reaction

Total RNAs were extracted by the TRIzol reagent (Invitrogen). In brief, cells seeded in 6-well plate were lysed with 1 mL of TRIzol, thoroughly mixed with 0.2 mL of chloroform, and then centrifugated by 12,000 g for 15 min at 4℃. The upper aqueous phase containing RNA was transferred, and RNA was precipitated by adding 0.5 mL of isopropanol and incubating at room temperature for 15 min. The extracted RNAs were washed with 75% ethanol twice and then dissolved in RNase free water. The complemental DNAs were synthesized with 1 μg of total RNAs by using the High-Capacity cDNA Reverse Transcription Kit (Life Technology). Each real-time PCR was run in a 10 μL reaction system containing 5 μL of SYBR Green Master Mix (Roche), 0.4 μL of 10 mM forward and reverse primers, and 4.2 μL of diluted cDNA. The reactions were performed on an ABI StepOne™ RT-PCR instrument, and the amplification program was performed at 95 ℃ for 5 min, following by 40 cycles of denaturation at 95 ℃ for 15 s, annealing and extension at 60 ℃ for 30 s. All reactions were run in triplicate. For detection of mature miRNAs, the TaqMan MicroRNA assay kit (Applied Biosystems) was used. RT-PCR values of specific mRNA or miRNA were normalized to the RT-PCR values of *GAPDH* (for mRNAs, pri-miRNAs and pre-miRNAs) or U6 snRNA (for mature miRNAs) with the calculation of 2^−ΔΔCt^ method. The sequences of the forward and reverse PCR primers are: 5′-CTCTGCTCCTCCTGTTCGAC-3′ and 5′-GCGCCCAATACGACCAAATC-3′ for human *GAPDH*; 5′-CTCGCTTCGGCAGCACA-3′ and 5′-AACGCTTCACGAATTTGCGT-3′ for human U6; 5′-GAGGAAGAGTGGAAGGTAGCCA-3′ and 5′-AGCATCGTTATCCATCATCACC-3′ for human pri-miR-103a-2; 5′-GTGCTGCCTTGTAGCATTCA-3′ and 5′-CCCTGTACAATGCTGCTTGA-3′ for human pre-miR-103a-2; 5′-TAGCCCTGTACAATGCTGCTT-3′ and 5′-TTACAGTGCTGCCTTGTTGC-3′ for human pre-miR-103b-1; 5′-CTTGTCCTCACGGTCCAGTT-3′ and 5′-CAGGAATCCCCATCTTAGCA-3′ for human pre-miR-145; 5′-AGTGCTCTCGTCTCCCCTCT-3′ and 5′-GGCGAACATACCTGTGACCT-3′ for human pre-miR-192; 5′-CTTGTGGCATGGAGTTCAAG-3′ and 5′-TCTGTGCTTTGATAGCCCTGT-3′ for human pre-miR-107; 5′-ATCGCCTTCACTGCCTTTTG-3′ and 5′-GTGCAGCATTTTCAGGGACA-3′ for human Dicer.

### RNA immunoprecipitation (RNA-IP)

Cells were crosslinked for 10 min with 1% formaldehyde at 37 ℃ and then incubated with 0.125 M glycine dissolved in PBS for 5 min at room temperature. Cells were washed twice with cold PBS and resuspended in RIPA buffer (50 mM Tris [pH 7.4], 150 mM NaCl, 1 mM EDTA, 0.1% SDS, 1% NP-40, 0.5% sodium deoxycholate, and 0.5 mM DTT) with proteinase inhibitor cocktail and RNase inhibitors. The lysates were sonicated and centrifugated at 12,000 rpm for 10 min and split into two parts, of which 5% of the lysates for input analysis and the rest for immunoprecipitation analysis. The specific antibodies and beads were added into the lysates followed by incubation at 4 ℃ for 4 h. Beads were successively washed twice with RIPA buffer, 4 times with 1 M RIPA buffer (50 mM Tris [pH 7.4], 1 M NaCl, 1 mM EDTA, 0.1% SDS, 1% NP-40, and 0.5% sodium deoxycholate), and twice with RIPA buffer. The beads were resuspended in RIPA buffer and treated with proteinase K at 45 ℃ for 45 min. The RNA was isolated by using TRIzol™ LS reagent (Cat.10296010, Thermo Fisher Scientific) and resuspended in buffer with DNase I (10 U) to remove DNA. The isolated RNA was dissolved in water and used for cDNA synthesis reaction. QRT-PCR reactions were then performed using specific primers. The qRT-PCR values of the pre-miRNAs were normalized to the qRT-PCR values of the adjusted pre-miRNA input with the calculation of 2^−(Ct (IP)−Ct(input))^. The data were presented as the percentages of the input.

### In vitro RNA synthesis and GST pull-down assay

RNA synthesis was performed by in vitro transcription reaction using MAXIscript™ Transcription kit (Ambion) with linear pcDNA3.1-pre-miR-103a-2 vector as the template, and synthesized RNA was purified by the Qiagen RNeasy kit. GST-RXRα and GST-tagged mutants (AB, DBD and LBD) were expressed in *E.coli* and pulled down by Glutathione Sepharose beads. Protein-bound beads were washed 4 times with washing buffer (10 mM Tris–HCl [pH 7.6], 0.5 M LiCl, and 0.1% Triton X-100) at 4 ℃ for 5 min, and then washed with binding buffer (20 mM Tris–HCl [pH 7.6], 0.1 M KCl, 0.1% Tween 20, and 0.1% Triton) for 10 min at 4 ℃. Beads with bound proteins (30 pmol) were resuspended in 100 μL binding buffer and incubated with 25 μg tRNA, 1 ng poly-[dI-dC] and 2 μL RNase inhibitor for 10 min before the incubation with ~ 5 pmol (250 ng) transcribed pre-miR-103a-2. After incubation for 1 h at 4 ℃, beads were washed 4 times with binding buffer. RNA was eluted by elution buffer (1% SDS and 150 mM NaCl) at room temperature and purified with TRIzol™ LS reagent. Eluted RNA was resuspended in water and used as templates for reverse transcription reaction followed by real-time PCR.

### RXRE reporter assay and mammalian one hybrid assay

For RXRE reporter assay, HEK293T cells seeded in 48-well plates at 2 × 10^4^ cells/well density were transiently transfected with pGL6-TA-RXRE-luciferase (50 ng), pCMV-Myc-RXRα (50 ng), pCMV-Renilla (2 ng), pcDNA3.1-pre-miR-103a-2 or mutant expression plasmids (50—200 ng). For mammalian one hybrid assay, HEK293T cells seeded in 48–well plates were transiently transfected with pG5-luciferase (50 ng), pBind-RXRα-LBD (50 ng), pcDNA3.1-pre-miR-103a-2 or mutant expression plasmids (50—200 ng). Twenty-four hours after transfection, cells were treated with 9-cis-RA for 12 h. Cells were lysed and luciferase activity was detected by using the Dual-Luciferase Reporter Assay System (Promega). The values of firefly luciferase activity were normalized to the values of renilla luciferase activity to obtain the relative luciferase activity.

### Western blot assay

Cells were lysed with the lysis buffer (50 mM Tris [pH 7.4], 150 mM NaCl, 1% NP-40, and 0.5% sodium deoxycholate including protease inhibitor and phosphatase inhibitor). The protein concentrations of the cell lysates were quantified by the BCA Protein Assay Kits (Thermo Scientific, Cat.23227). Proteins (15 μg of each well) along with protein ladder (Thermo Scientific, Cat.26616) were separated by Sodium dodecyl-sulfate polyacrylamide gel electrophoresis (SDS-PAGE) prior to transferring to polyvinylidene difluoride (PVDF) membranes (Millipore, MA, USA). The membranes were blocked with 5% skimmed milk in TBST (50 mM Tris–HCl [pH 7.4], 150 mM NaCl and 0.1% Tween 20) for 1 h, and then incubated with the primary antibodies over night at 4 ℃. The dilution ratios of antibodies used for incubation were described in the section of Reagents and antibodies. After washed with TBST (10 min, three times), the membranes were incubated with the secondary antibodies (goat anti-mouse or anti-rabbit lgG-HRP-conjugated, 1:5000) for 1 h at room temperature, and then washed with TBST three times (10 min/each time). The chemiluminescence signal was produced by using enhanced chemiluminescence (ECL) detection system (Advansta, Cat.K-12045-D50). In brief, membranes were incubated with the enhanced chemiluminescence reagent for 10 s, and the chemiluminescent signal was detected and recorded by exposure of the membranes to an X-ray film (BYSH, Shanghai, China).

### Nuclear and cytoplasmic fractionation

Cells seeded in 10-cm dishes were transfected with Myc-RXRα expression plasmids (2 μg). After 36 h of transfection, cells were collected by scraping and washed once with cold PBS. Cells were then resuspended in 200 μL of lysis buffer (10 mM Hepes [pH 7.9], 10 mM KCl, 1.5 mM MgCl_2_, 0.2 mM EDTA, 0.1 mM EGTA, and 1 mM DTT) with protease inhibitor cocktail and RNase inhibitors for 8 min, followed by adding 12 μL of 10% (vol/vol) NP-40 and shaking for 10 s. Cell lysates were then centrifuged at 12,000 g for 30 s at 4 ℃, and the supernatant was collected as the cytoplasmic fraction. The remaining pellets were washed three times with cold PBS and collected as nuclear fraction. The nuclear and cytoplasmic RNA were extracted respectively with TRIzol™ LS reagent according to the manufacturer’s instructions.

### Wound healing assay

Cells seeded in 12-well plates at the density of 2 × 10^5^ cells/mL were transfected with the plasmids and the miRNA mimics or inhibitors. After 24 h, cells reached about 90% confluence and wounds were generated by scratching using a 10-μL pipette tip. Cells were washed with PBS once and cultured in the complete culture media. Images were acquired immediately following media replacement (T0) using Leica DMi8 microsystem. Images were captured again after 24 h incubation (T24). Wound areas were measured using ImageJ software, and the wound healing rates were determined by normalization of the areas at T24 to the areas at T0.

### Statistical analysis

Data are presented as mean ± SEM, and the statistical significance are shown in the figure legends. Statistical analyses were performed by Student′s t test and one-way ANOVA. *p* < 0.05 (*) was considered as statistically significant, *p* < 0.01 (**) as highly significant, *p* < 0.001 (***) and *p* < 0.0001 (****) as extremely significant, and ns as not significant. All data were repeated by at least three independent experiments.

### Supplementary Information

**Additional file 1: Figure S1.** The purity of the proteins used in GST pull-down assays and the immunoprecipitation efficiency of the Flag antibody. **Figure S2.** Theoretical ΔG values and predicted secondary structure of pre-miR-103a-2 and its mutants. **Figure S3.** The efficiency of the cellular fractionation assay and the immunoprecipitation of XPO5 protein.

**Additional file 2.** The individual data values for Fig. 1a-d; Fig. 2b-d; Fig. 3b-d; Fig. 4a-e; Fig. 5a-d; Fig. 6a, c, and e; Fig. 7a and b.

**Additional file 3.** Original western blot and Coomassie blue protein staining data.

## Data Availability

All data generated or analyzed during this study are included in this published article and its supplementary information files. The datasets used and/or analyzed during the current study are available from the corresponding author on reasonable request.

## Abbreviations

miRNAs: MicroRNAs
RXRα: Retinoid x receptor alpha
pri-miRNAs: Primary miRNAs
pre-miRNA: Precursor miRNA
XPO5: Exportin-5
RXRE: Retinoid x response element
DBD: DNA-binding domain
LBD: Ligand-binding domain
RARs: Retinoic acid receptors
PPARs: Peroxisome proliferator-activated receptors
VDR: Vitamin D receptor
TRs: Thyroid hormone receptors
ERα: Estrogen receptor alpha
AR: Androgen receptor
RISC: RNA-induced silencing complex
