# Supplementary material for: Nuclear receptor RXRα binds the precursor of miR-103 to inhibit its maturation
Source: BMC Biol. 2023 Sep 21;21:197. doi: 10.1186/s12915-023-01701-3 (PMC10512521; doi:10.1186/s12915-023-01701-3)
Supplement: Supplementary file 1 — Additional file 1: Figure S1. The purity of the proteins used in GST pull-down assays and the immunoprecipitation efficiency of the Flag antibody. Figure S2. Theoretical ΔG values and predicted secondary structure of pre-miR-103a-2 and its mutants. Figure S3. The efficiency of the cellular fractionation assay and the immunoprecipitation of XPO5 protein. [file 12915_2023_1701_MOESM1_ESM.docx]

Additional file 1

**Nuclear receptor RXRα binds the precursor of miR-103 to inhibit its maturation**

Xiaohong Ye^1,2†^, Yun Yang^1†^, Jiayue Yao^1^, Mo Wang^1^, Yixin Liu^1^, Guobin Xie^1^, Zhiping Zeng^1,2^, Xiao-kun Zhang^1,2^, Hu Zhou^1,2*^

^†^Xiaohong Ye and Yun Yang contributed equally to this work

^*^Correspondence: Hu Zhou, [huzhou@xmu.edu.cn](mailto:huzhou@xmu.edu.cn)

^1^School of Pharmaceutical Sciences, Fujian Provincial Key Laboratory of Innovative Drug Target Research, Xiamen University, Xiamen, Fujian 361102, China

^2^High Throughput Drug Screening Platform, Xiamen University, Xiamen, Fujian 361102, China

Additional file 1: Figure S1 to S3


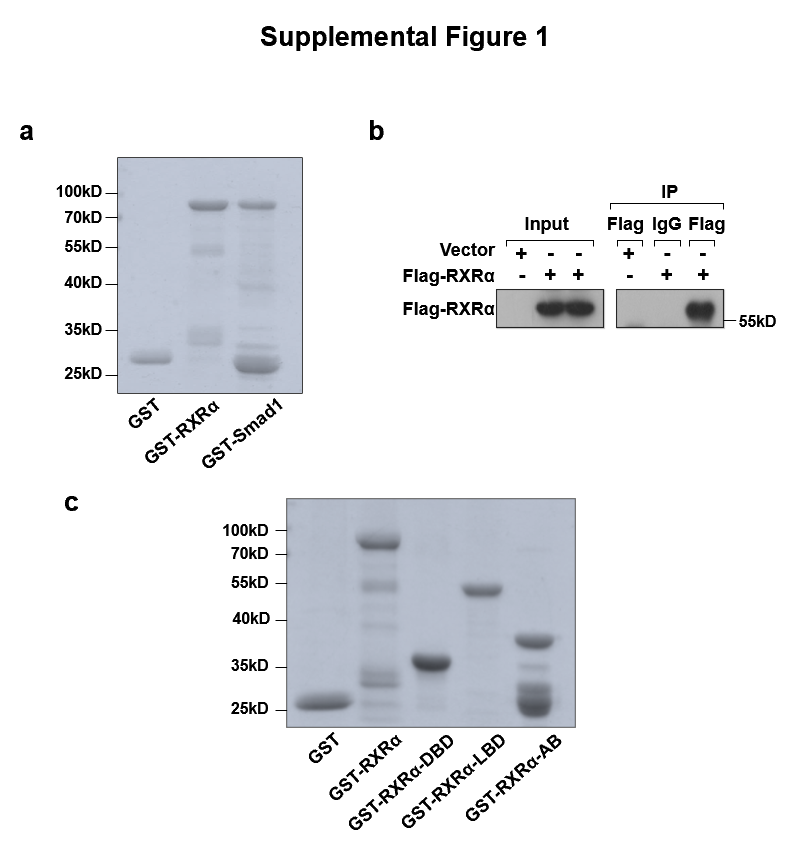


**Figure S1.** Analysis of the purity of the proteins used in GST pull-down assays and the immunoprecipitation efficiency of the Flag antibody. **a** The purity of GST, GST-RXRα, GST-Smad1 proteins purified from *E.coli* were analyzed by SDS-PAGE and Coomassie Blue staining. **b** The lysates from stable control and stable Flag-RXRα-expressed AD293 cells were subjected to immunoprecipitation with non-specific IgG or anti-Flag antibody, followed by western blot analysis. **c** The purity of GST, GST-RXRα and GST-tagged RXRα mutant proteins purified from *E.coli* were analyzed by SDS-PAGE and Coomassie Blue staining.

**
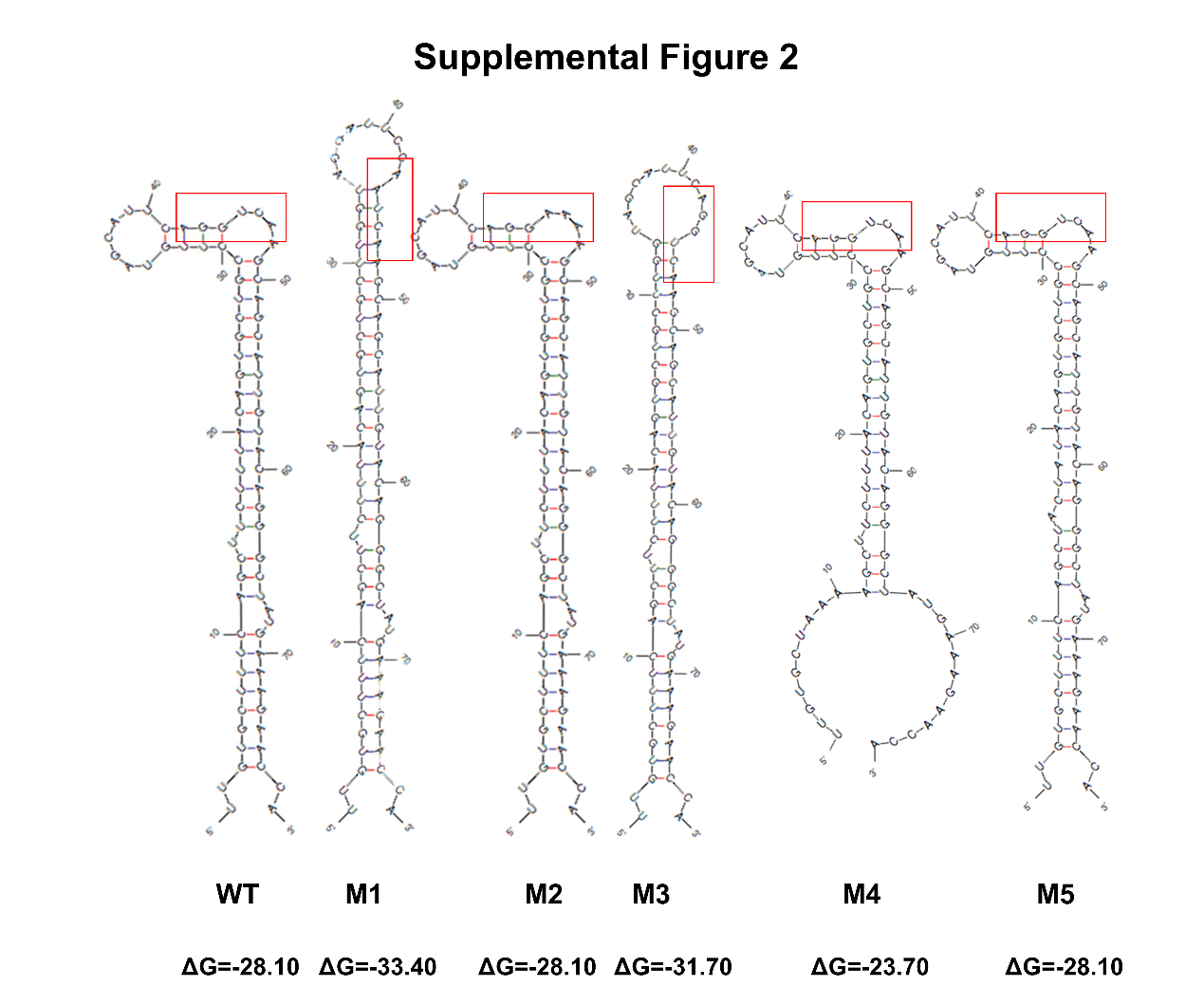
**

**Figure S2.** The theoretical ΔG values and the predicted secondary structures of pre-miR-103a-2 and its mutants. The secondary structures of premiR-103a-2 and its mutants were predicted by mFold v3.2. Theoretical ΔG values (kcal/mol) were calculated using two algorithms (i) DINAMelt server by UNAFold: update of mFold v3.0 (http://dinamelt.bioinfo.rpi.edu/quikfold.php) and (ii) mFold v3.2 (<http://mfold.bioinfo.rpi.edu/cgi-bin/rna-form1.cgi>). R-RXRE is framed by red border box.


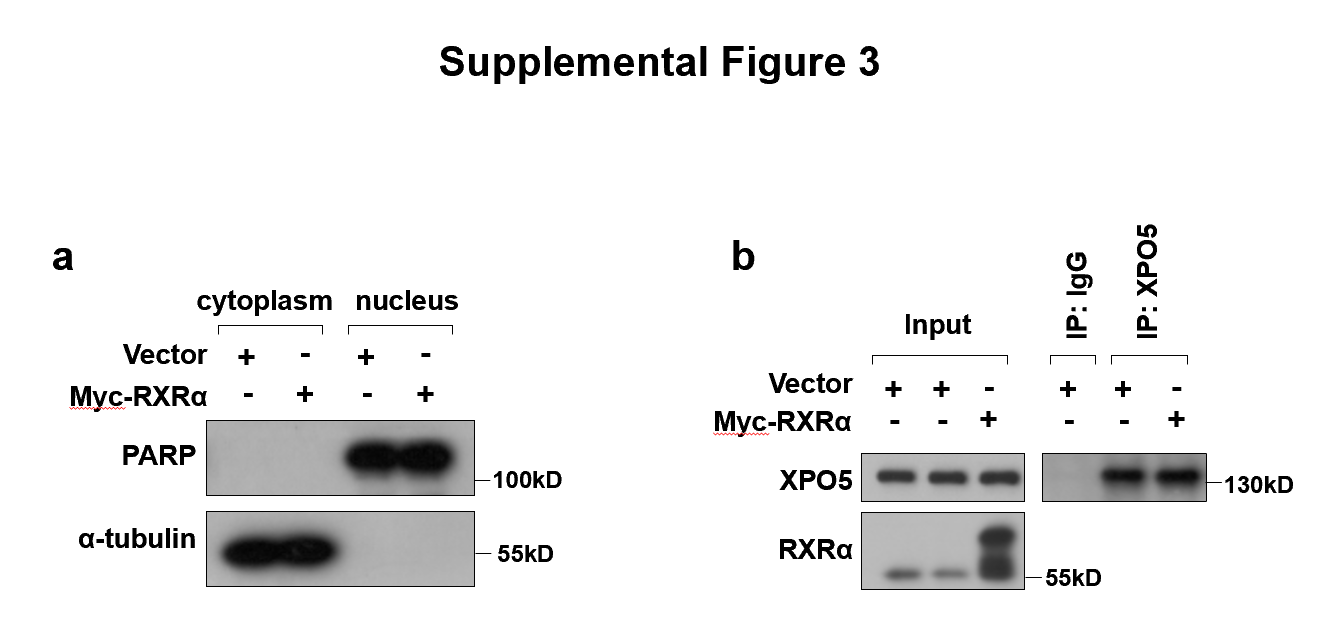


**Figure S3.** Analysis of the efficiency of the cellular fractionation assay and the immunoprecipitation of XPO5 protein. **a** The efficiency of nuclear and cytoplasmic fractionation was analyzed by western blot using anti-PARP and anti-α-tubulin antibodies. **b** HeLa cells were transfected with control or Myc-RXRα expression plasmids. Cell lysates were used for immunoprecipitation with anti-XPO5 antibody or non-specific IgG, followed by western blot analysis.
