# Supplementary material for: Nuclear receptor RXRα binds the precursor of miR-103 to inhibit its maturation
Source: BMC Biol. 2023 Sep 21;21:197. doi: 10.1186/s12915-023-01701-3 (PMC10512521; doi:10.1186/s12915-023-01701-3)
Supplement: Supplementary file 3 — Additional file 3. Original western blot and Coomassie blue protein staining data. [file 12915_2023_1701_MOESM3_ESM.pptx]

## Slide 1
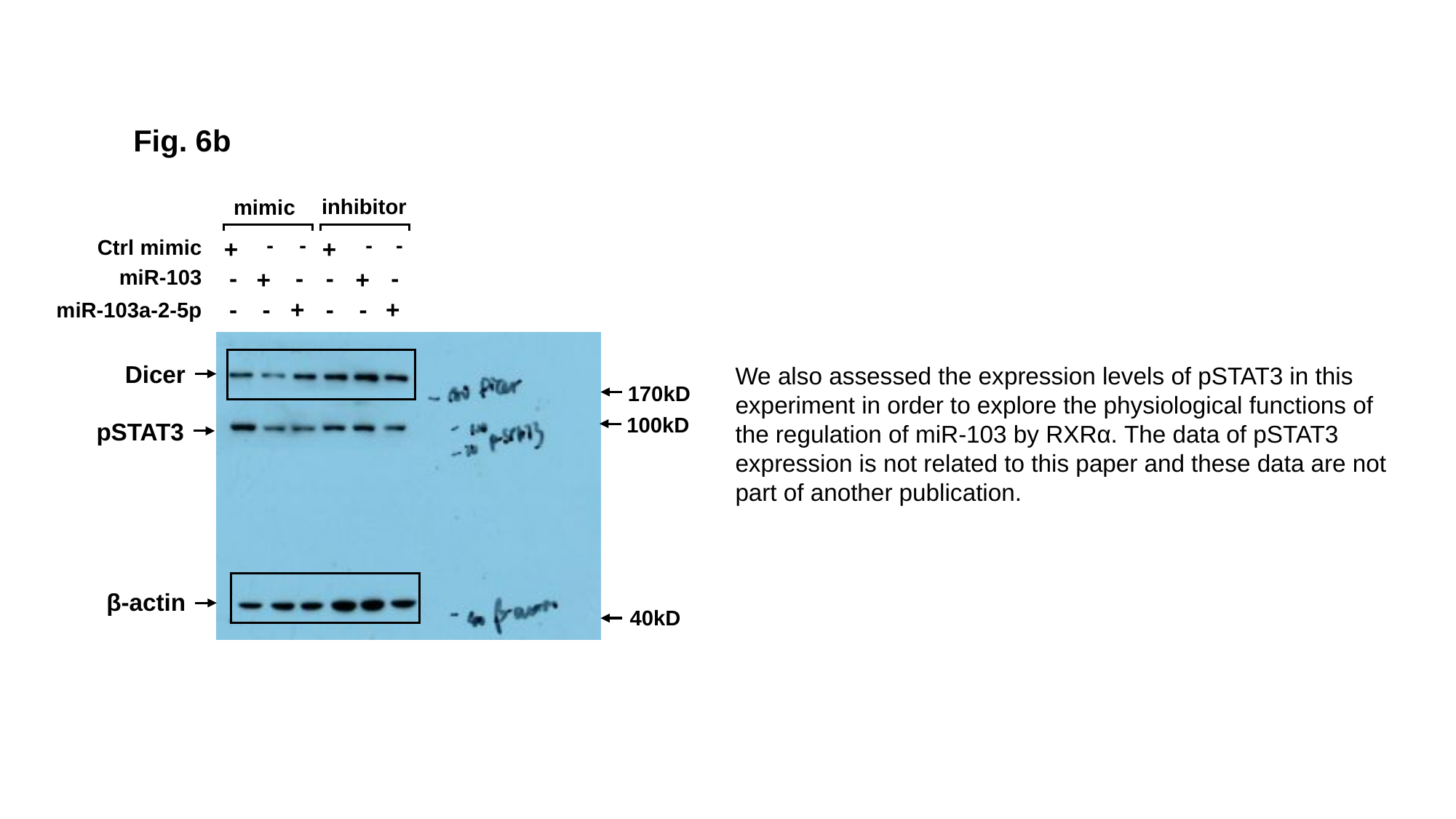

Fig. 6b
inhibitor
mimic
-
-
-
-
Ctrl mimic
miR-103
miR-103a-2-5p
+
+
-
-
-
-
+
+
-
-
-
-
+
+
Dicer
We also assessed the expression levels of pSTAT3 in this experiment in order to explore the physiological functions of the regulation of miR-103 by RXRα. The data of pSTAT3 expression is not related to this paper and these data are not part of another publication.
170kD
100kD
pSTAT3
β-actin
40kD

## Slide 2
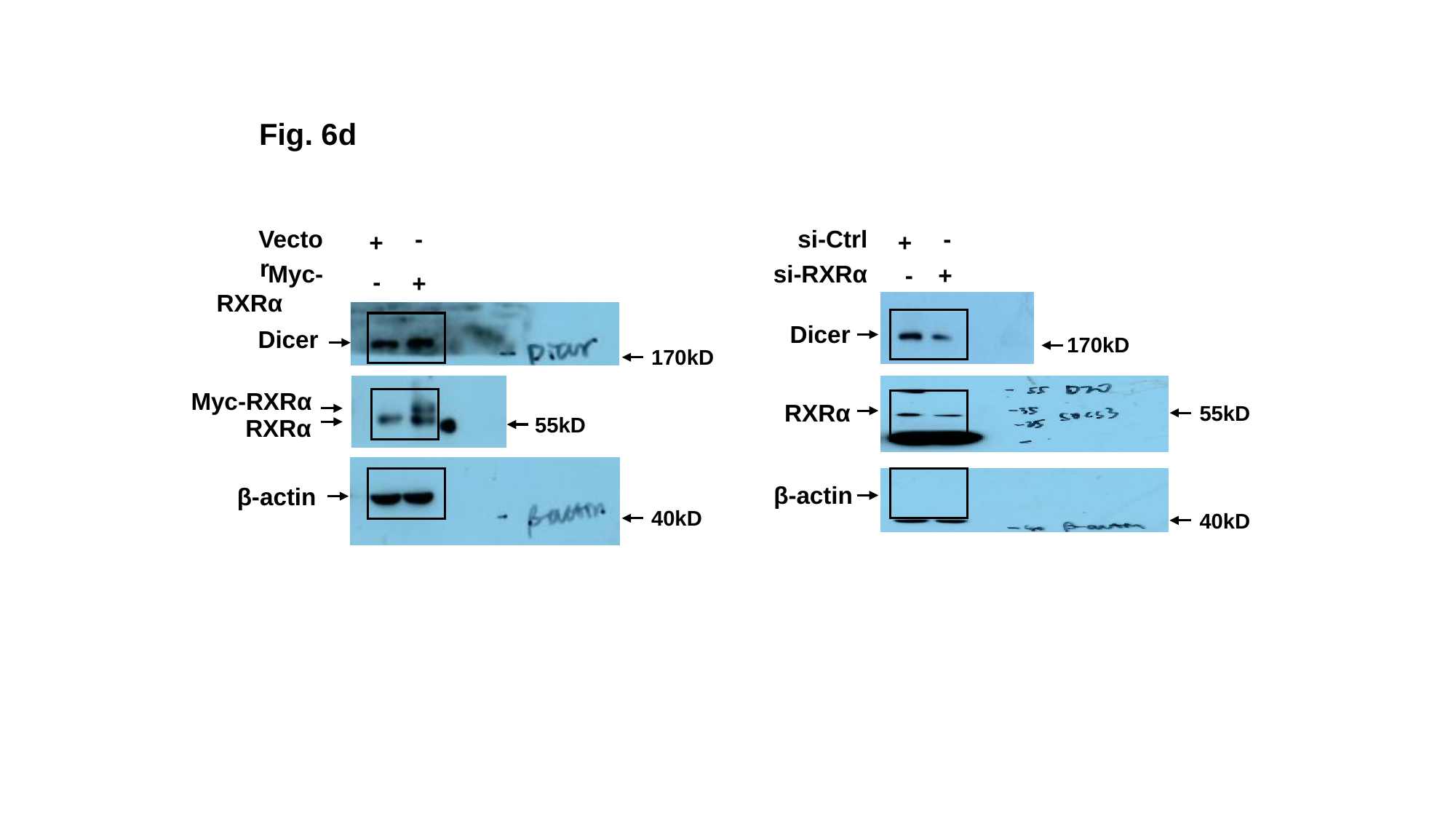

Fig. 6d
si-Ctrl
si-RXRα
Vector
Myc-RXRα
-
-
+
+
-
+
-
+
Dicer
Dicer
170kD
170kD
Myc-RXRα
RXRα
55kD
55kD
RXRα
β-actin
β-actin
40kD
40kD

## Slide 3
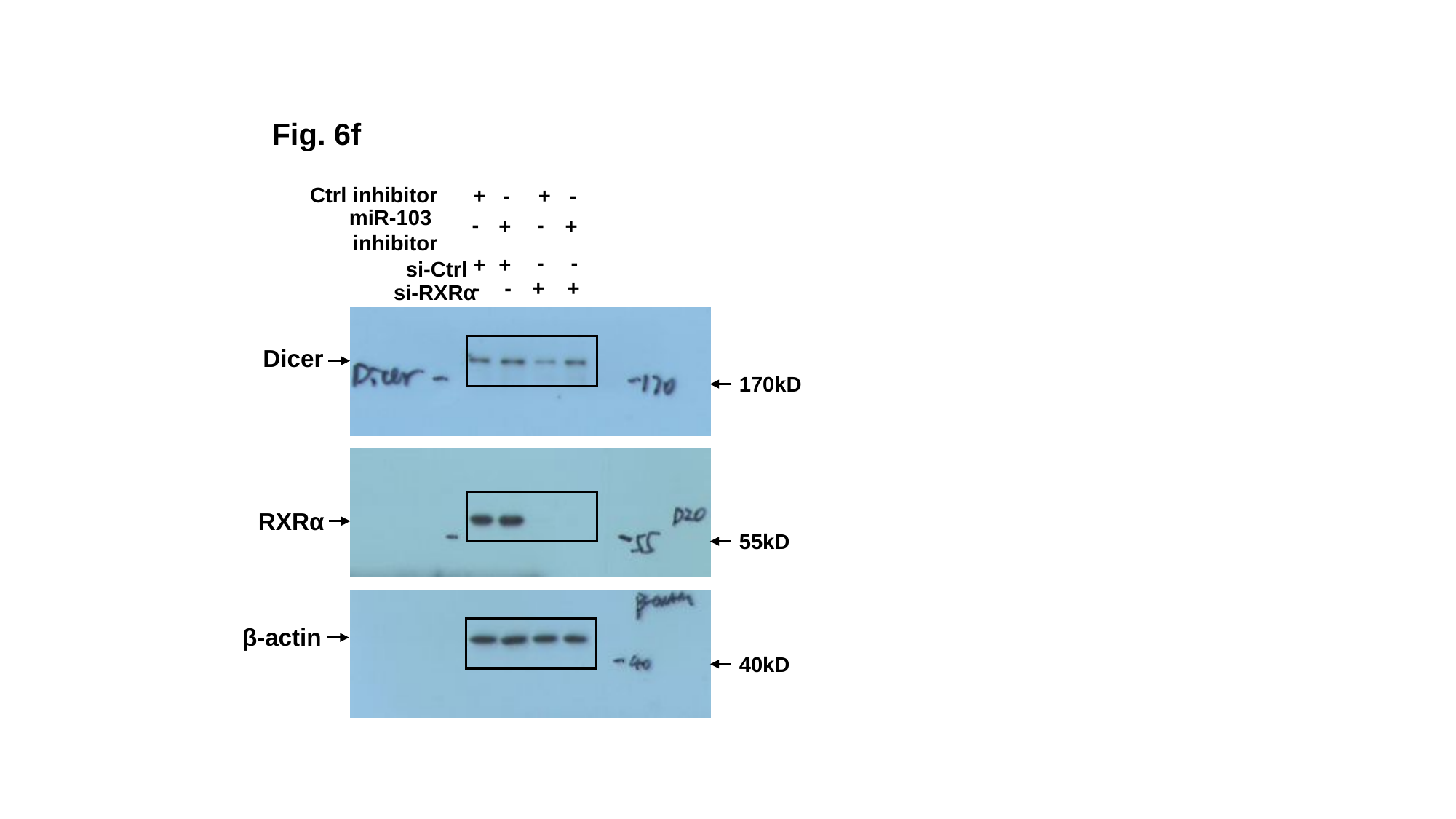

Fig. 6f
Ctrl inhibitor
miR-103
inhibitor
si-Ctrl
si-RXRα
+
+
-
-
-
-
+
+
-
-
+
+
-
-
+
+
Dicer
170kD
RXRα
55kD
β-actin
40kD

## Slide 4
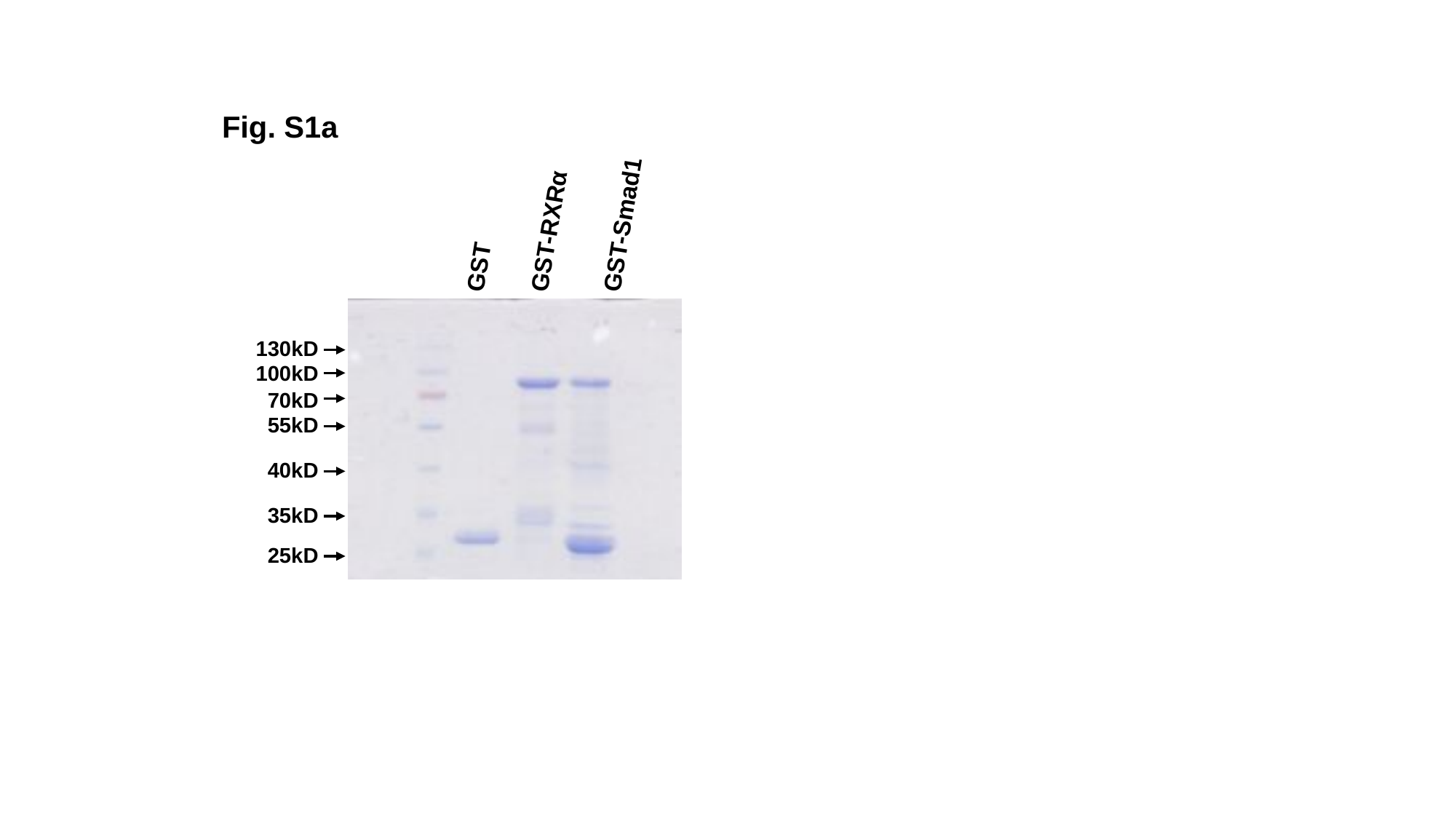

Fig. S1a
GST-Smad1
GST-RXRα
GST
130kD
100kD
70kD
55kD
40kD
35kD
25kD

## Slide 5
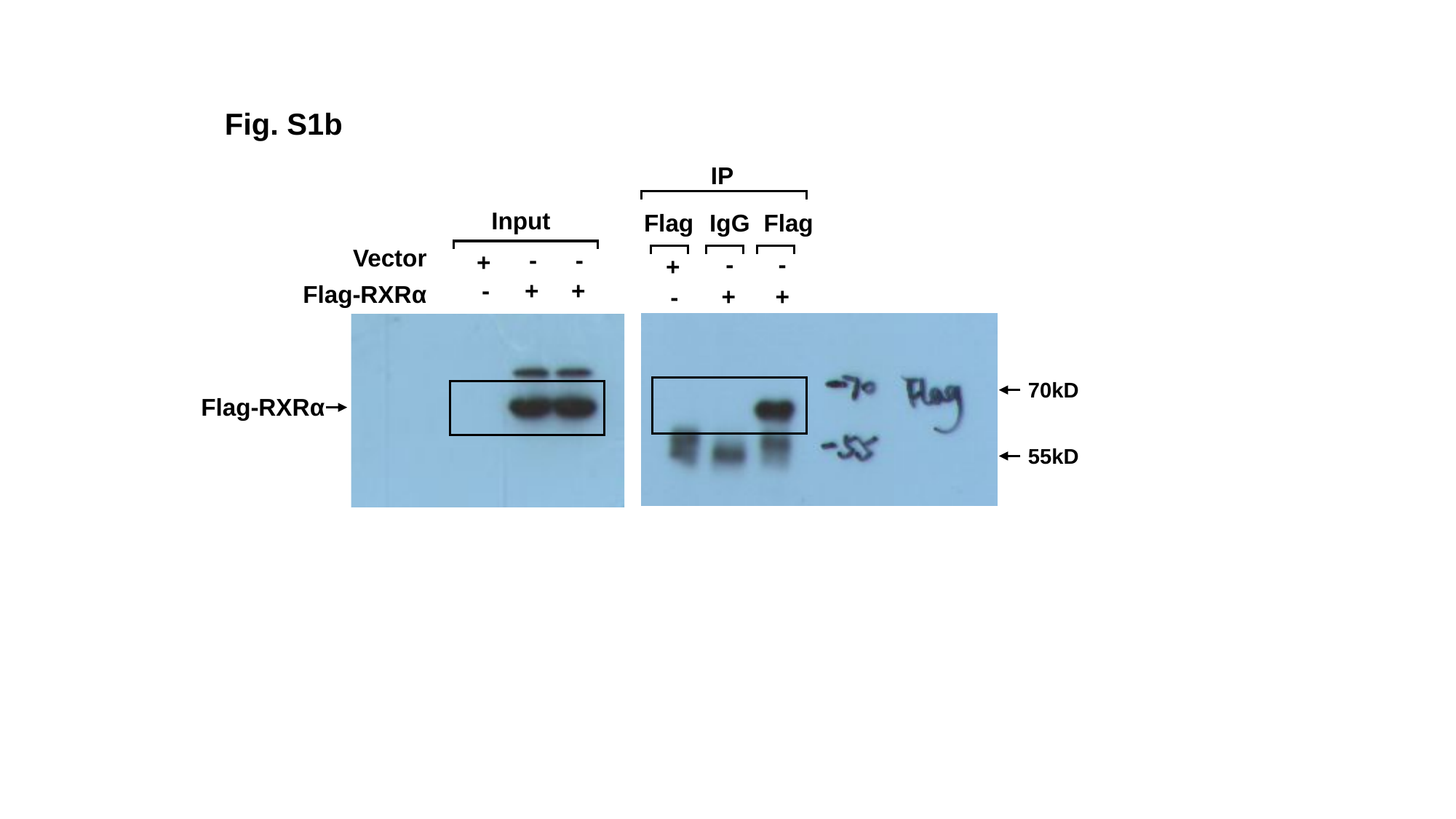

Fig. S1b
IP
Flag
 IgG
Flag
-
-
+
+
+
-
Input
Vector
-
-
+
+
+
-
Flag-RXRα
70kD
Flag-RXRα
55kD

## Slide 6
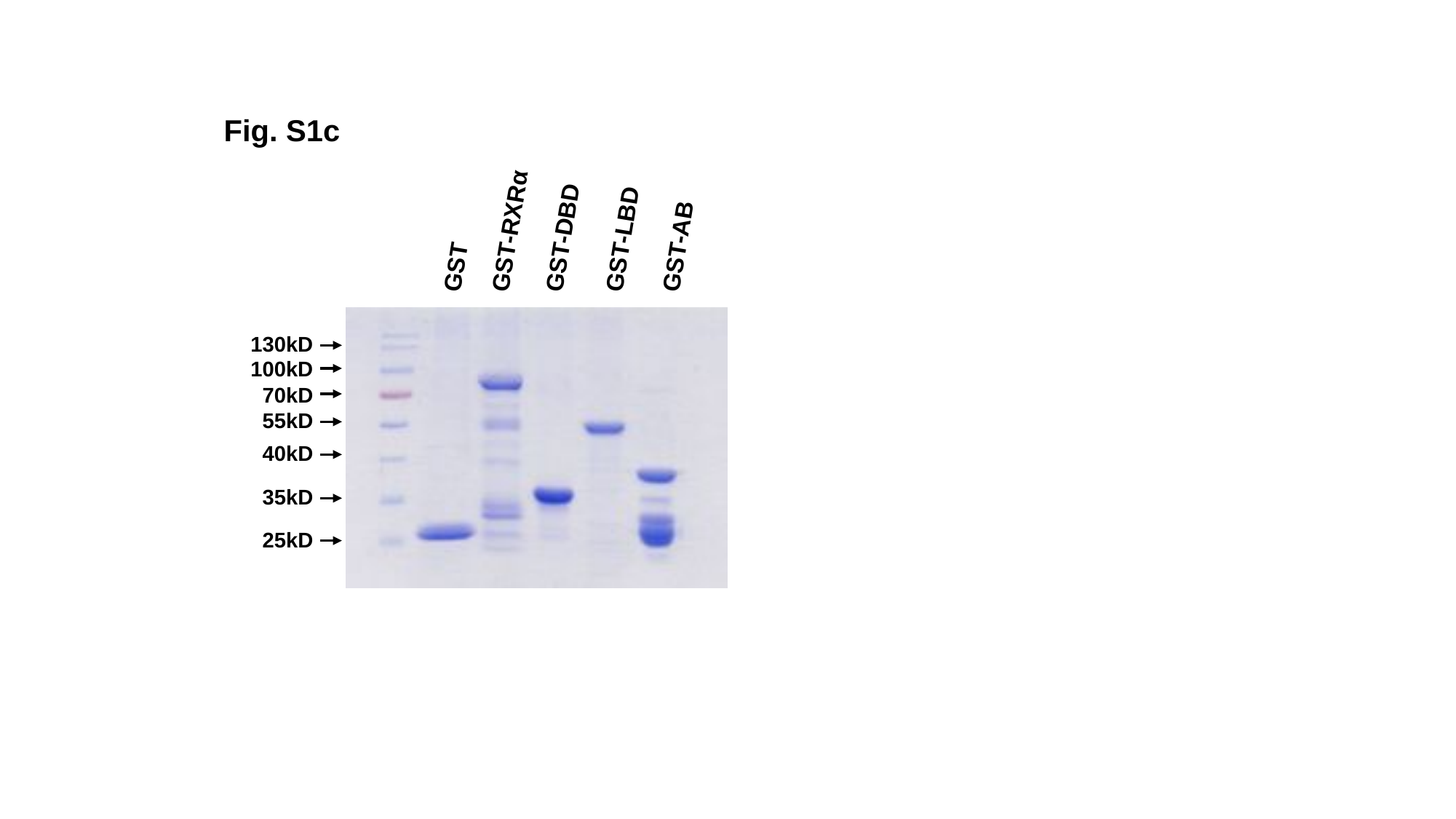

Fig. S1c
GST-RXRα
GST-LBD
GST-DBD
GST-AB
GST
130kD
100kD
70kD
55kD
40kD
35kD
25kD

## Slide 7
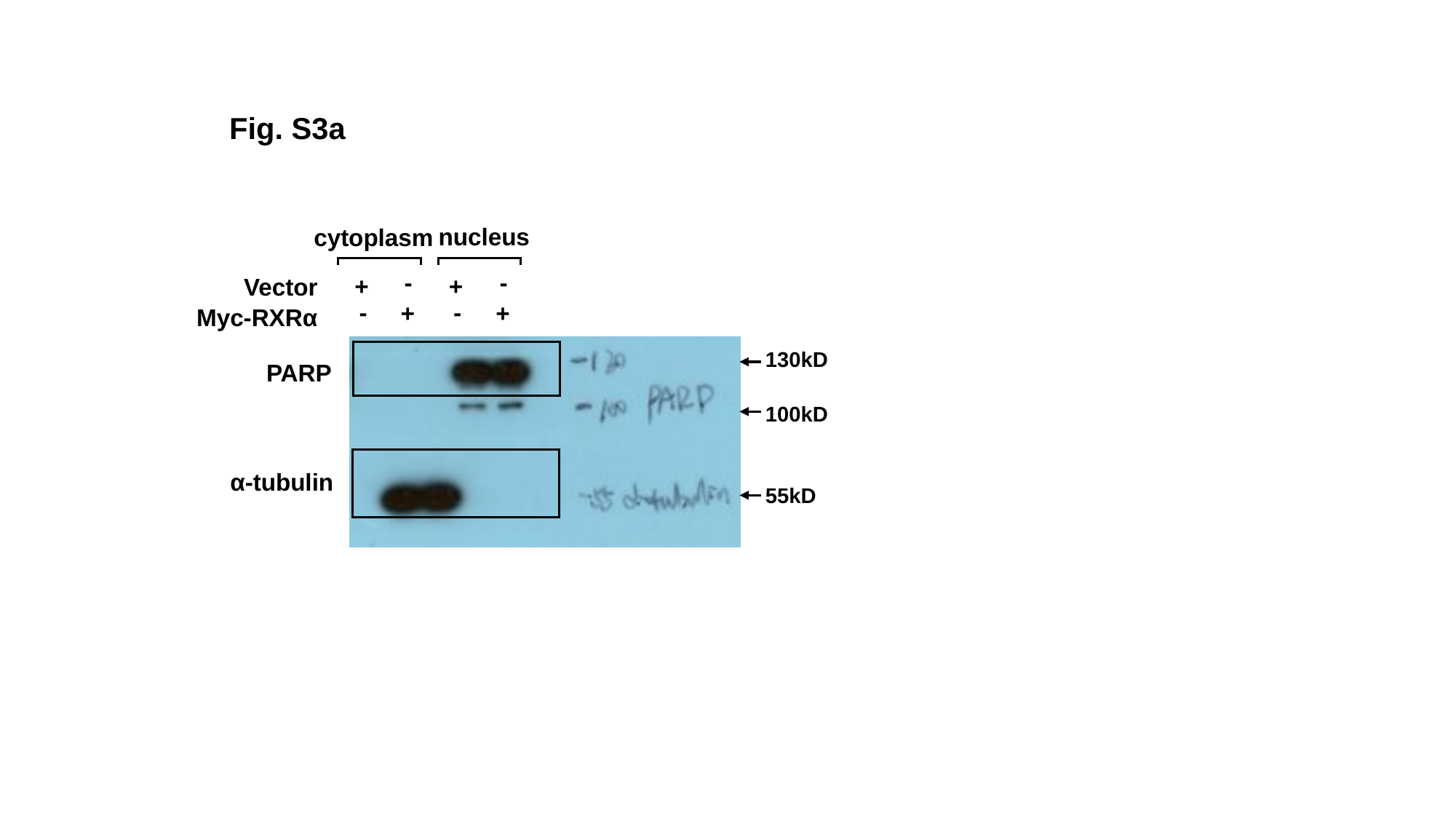

Fig. S3a
nucleus
cytoplasm
Vector
Myc-RXRα
-
-
+
+
-
-
+
+
130kD
PARP
100kD
α-tubulin
55kD

## Slide 8
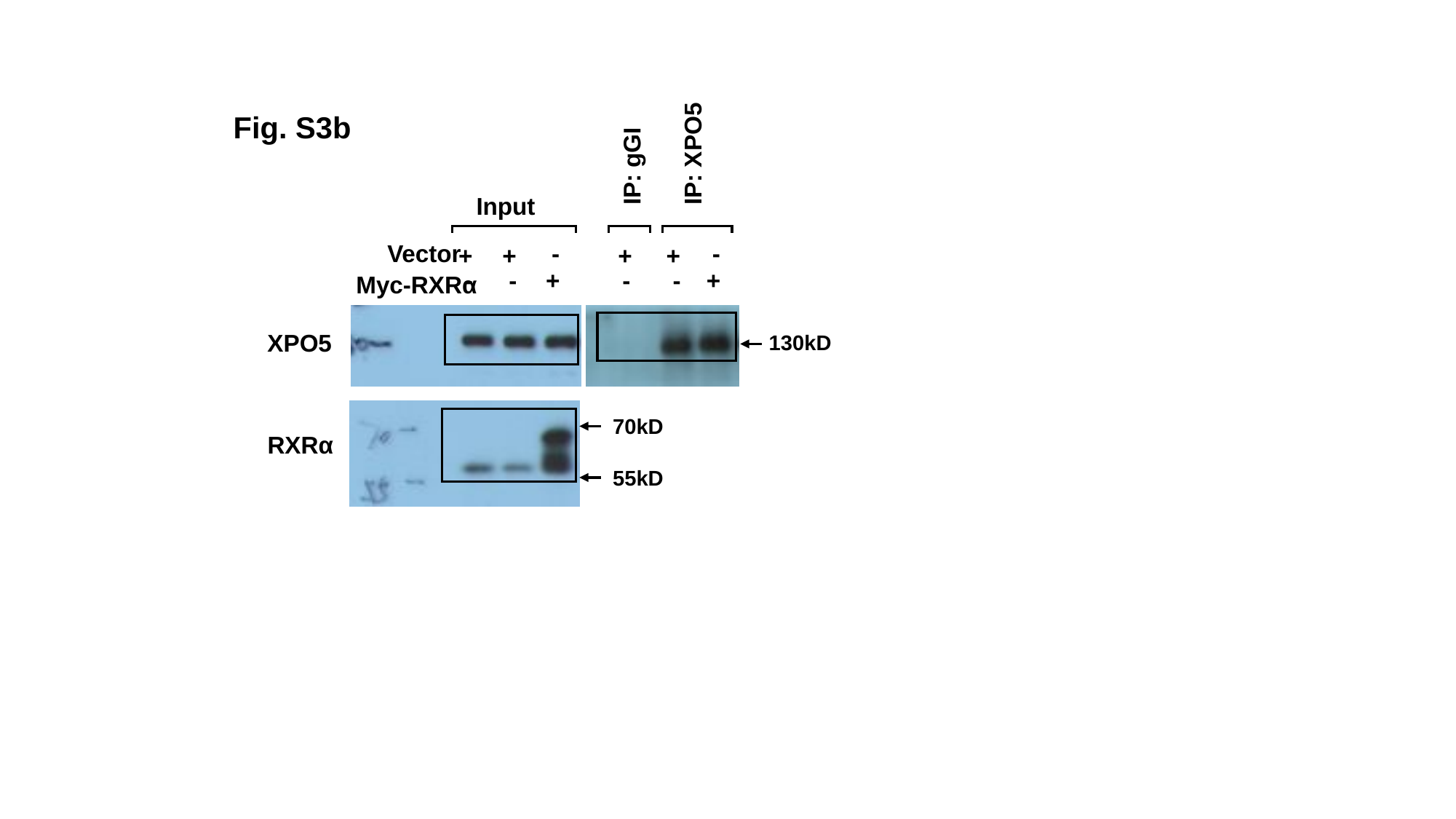

Fig. S3b
IP: XPO5
IP: gGI
Input
Vector
-
+
-
+
+
-
+
-
+
-
+
-
Myc-RXRα
XPO5
130kD
70kD
RXRα
55kD
